# Supplementary material for: Trends in actinide electronic structure revealed from asymmetric, isostructural transuranic metallocenes
Source: Commun Chem. 2025 Sep 26;8:280. doi: 10.1038/s42004-025-01646-4 (PMC12475471; doi:10.1038/s42004-025-01646-4)
Supplement: Supplementary file 2 — Supplementary Information [file 42004_2025_1646_MOESM2_ESM.pdf]

Electronic Supporting Information

Trends in actinide electronic structure revealed from asymmetric, isostructural transuranic metallocenes

Cambell S. Conour,[#1, 2] Mikaela Mary F. Pyrch,[#1] Nicholas Katzer,[1, 2] Asmita Sen,[3] Joshua Woods,[2] Jochen Autschbach*,[3] Polly L. Arnold*[1, 2]

[#]Authors contributed equally to this work

Table of Contents

S1. Additional Synthesis Notes S2

S2. NMR Spectra S4

S3. Crystallographic Data S9

S4. UV-VIS Spectroscopy S18

S5 Additional Characterization S22

S5.1. IR spectroscopy S22

S5.2 Fluorescence spectroscopy S23

6. Computational Analysis S24

S7. References S38

S1. Supplementary Methods

($K_2[1,4\text{-}(Ph_3Si)_2C_8H_6]_2(C_7H_8)$): Inside a glovebox, a 50 mL flask was charged with a large PTFE stir bar, 3.07 g H_2COT^{big} (4.93 mmol), and toluene (5 mL). To this suspension, a solution of 2.08 g KHMDS (10.4 mmol, 2.1 equivalents) in toluene (15 mL) was added dropwise over the course of 10 minutes with vigorous stirring. The brown mixture was stirred for 3 days, after which the solids were collected on a medium-fitted filter and washed with 5 mL portions of hexanes until the filtrate ran clear (typically 3 to 5 washes). The isolated bright yellow solid was dried under reduced pressure for 4 hours to give K_2COT^{big}. Yield: 2.54 g (65 %).

X-ray quality crystals were grown by vapor diffusion of hexanes into a toluene solution of K_2COT^{big}.

^{1}H NMR (600 MHz, d_8-THF): δ 7.69 – 7.63 (m, 12H, o-Ph), 7.21 – 7.04 (m, 23H, m/p-Ph and Toluene), 6.43 (s, 2H, COT-H2/3), 6.38 (dd, J = 8.1, 3.6 Hz, 2H, COT-H6/7), 6.06 (dd, J = 8.1, 3.6 Hz, 2H, COT-H5/8), 2.31 (s, 3H, Toluene-CH_3).

^{13}C NMR (151 MHz, d_8-THF): δ 143.13 (ipso-Ph), 138.61, 137.77 (o-Ph), 135.93, 130.71, 129.84, 129.08, 128.67, 128.25 (p-Ph), 127.57 (m-Ph), 126.21, 103.05 (COT-C6/7), 102.16 (COT-C2/3), 94.84 (COT-C5/8), 82.46 (COT-C1/4), 21.65 (Tol-CH_3).

^{29}Si NMR (119 MHz, d_8-THF): δ -7.02.

UV-vis-NIR λ_{max} (ε): 374 nm (5040 $M^{-1}cm^{-1}$), 295 nm (8820 $M^{-1}cm^{-1}$).

FTIR: 3064 (w), 3053 (w), 3017 (w), 3003 (w), 2962 (w), 1548 (w), 1427 (m), 1259 (w), 1201 (w), 1119 (m), 1101 (s), 1051 (m), 1029 (w), 997 (w), 987 (w), 968 (w), 931 (m), 903 (w), 868 (w), 768 (w), 732 (s), 696 (vs), 680 (m), 633 (w), 620 (w), 573 (vs), 545 (m), 530 (s), 506 (s), 488 (s), 479 (s), 467 (m), 453 (m), 536 (m), 426 (w), 413 (w).

Analysis for $C_{51}H_{44}K_2Si_2$: Expected: C 77.41 %, H 5.60 %. Found: C 77.11 %, H 5.59 %.

1Th ($Th^{IV}[1,4\text{-}(Ph_3Si)_2C_8H_6]_2(C_7H_8)$): An orange solution of K_2COT^{big} (101 mg, 128 μmol, 2.5 equiv) in THF (2 mL) was added dropwise to a stirring solution of $ThCl_4(DME)_2$ (28 mg, 51 μmol) in THF (1 mL), giving a bright yellow mixture. After stirring for 4 hours, 3 mL of hexanes was added dropwise, and the mixture stirred for an additional 30 minutes. The mixture was centrifuged and the yellow supernatant was decanted and discarded and the remaining yellow solids were washed three times with 2 mL of 1:1 THF:hexanes to remove excess K_2COT^{big}. The resulting beige solids were then extracted into 3 mL of toluene and immediately filtered to give a bright yellow solution. Removal of the solvent under reduced pressure gave $Th^{IV}[1,4\text{-}(Ph_3Si)_2C_8H_6]_2$, **1Th**, as a bright yellow powder (54 mg, 34 μmol, 68 % yield). Single crystals suitable for X-ray diffraction were obtained by vapor diffusion of hexanes into a toluene solution of **1Th**, yielding **1Th·tol**. Likewise, crystalline **1Th·bz** ($Th[1,4\text{-}(Ph_3Si)_2C_8H_6]_2(C_6H_6)$) was obtained by vapor diffusion of hexanes into a benzene solution of **1Th.**

1U ($U^{IV}[1,4\text{-}(Ph_3Si)_2C_8H_6]_2(C_7H_8)$): An orange solution of K_2COT^{big} (101 mg, 128 mol, 2.5 equiv) was added dropwise as a homogenous THF solution (2 mL) to a stirring solution of UCl_4 (19 mg, 51 μmol) in THF (1 mL). Immediately upon addition, the solution turned into a purple

suspension, presumed to be the half-sandwich intermediate, $U(COT^{big})Cl_2$. Within two minutes, the suspension became green. After stirring for 4 hours, the mixture was centrifuged. The yellow supernatant was decanted and discarded and the remaining green solids were washed with 1:1 THF:hexanes (3x1 mL) to remove excess K_2COT^{big}. The resulting green solids were then immediately extracted into 3 mL of toluene before filtering through a glass microfiber filter. Concentrating the resulting green filtrate yielded $U^{IV}[1,4\text{-}(Ph_3Si)_2C_8H_6]_2$, **1U**, as an emerald green powder (60 mg, 38 mol, 75 % yield). Single crystals suitable for X-ray diffraction were obtained by vapor diffusion of hexanes into a toluene solution of **1U**, yielding **1U·tol**. Likewise, crystalline **1U·bz** ($U[1,4\text{-}(Ph_3Si)_2C_8H_6]_2(C_6H_6)$) was obtained by vapor diffusion of hexanes into a benzene solution of **1U.**

1Np ($Np^{IV}[1,4\text{-}(Ph_3Si)_2C_8H_6]_2(C_7H_8)$): THF (200 µL) was added to a vial containing solid $NpCl_4(DME)_2$ (4.71 mg, 8.43 µmol) forming a homogenous, pink solution. This solution was then added dropwise to a 20 mL scintillation vial containing a solution of K_2COT^{big} (16.67mg, 21.06 µmol, 500 µL, 2.5 equiv), followed by additional rinses with THF (3x200 µL). Within five minutes, the solution became red-orange in color and after 16 hours, the solution was completely opaque and deeper red in color. This mixture was centrifuged for five minutes. The THF supernatant was decanted into a 4 mL vial while the red solids were dissolved into toluene (1.5 mL) and transferred into a separate 4 mL vial. Vapor diffusion of hexanes into the toluene mixture over 16 h afforded red, crystalline needles while vapor diffusion of hexanes into the corresponding THF solution led to the precipitation of a red powder. This red powder was then redissolved in approximately 1 mL of toluene and recrystallized by diffusion of hexanes into the toluene solution, again yielding red crystalline material. The supernatants were decanted off the crystalline material and the solids were dried, yielding $Np^{IV}[1,4\text{-}(Ph_3Si)_2C_8H_6]_2$, **1Np**, as a red solid (10.26 mg combined yield, 6.531 µmol, 77.52 %). Single crystals suitable for X-ray diffraction were obtained by vapor diffusion of hexanes into a toluene solution of **1Np**, yielding **1Np·tol**.

1Pu ($Pu^{IV}[1,4\text{-}(Ph_3Si)_2C_8H_6]_2(C_7H_8)$): THF (200µL) was added to a vial containing solid $PuCl_4(DME)_2$ (8.27 mg, 14.6 µmol) forming a pink solution. This solution was then added dropwise to a 20 mL scintillation vial containing K_2COT^{big} (25.4 mg, 32.1 µmol, 500 µL, 2.2 equiv). The solution was stirred for 24 hours, eventually forming a maroon suspension. The solution was then centrifuged for 5 minutes, yielding a yellow supernatant and a deep red powder. The powder was rinsed with additional THF (3 x 1 mL) to remove excess K_2COT^{big}. The remaining red solid was redissolved in 1 mL of toluene and $Pu^{IV}[1,4\text{-}(Ph_3Si)_2C_8H_6]_2$**, 1Pu**, was isolated as crystalline needles by vapor diffusion of hexanes. The supernatants were decanted off the crystalline material and the solids were dried, yielding **1Pu** as a maroon solid (7.4 mg, 4.7 µmol, 32% yield). Single crystals suitable for X-ray diffraction were obtained by vapor diffusion of hexanes into a benzene solution of **1Pu**, yielding **1Pu·bz** ($Pu^{IV}[1,4\text{-}(Ph_3Si)_2C_8H_6]_2(C_6H_6)$).

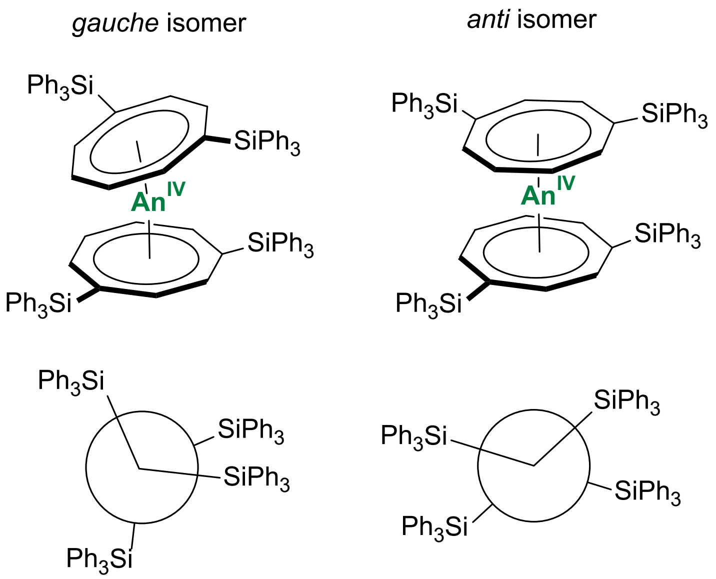

Figure S1 'Gauche' (observed in solid state) and 'anti' (proposed) isomers of **1An** with corresponding Newman projections (bottom).

S2. NMR Spectra

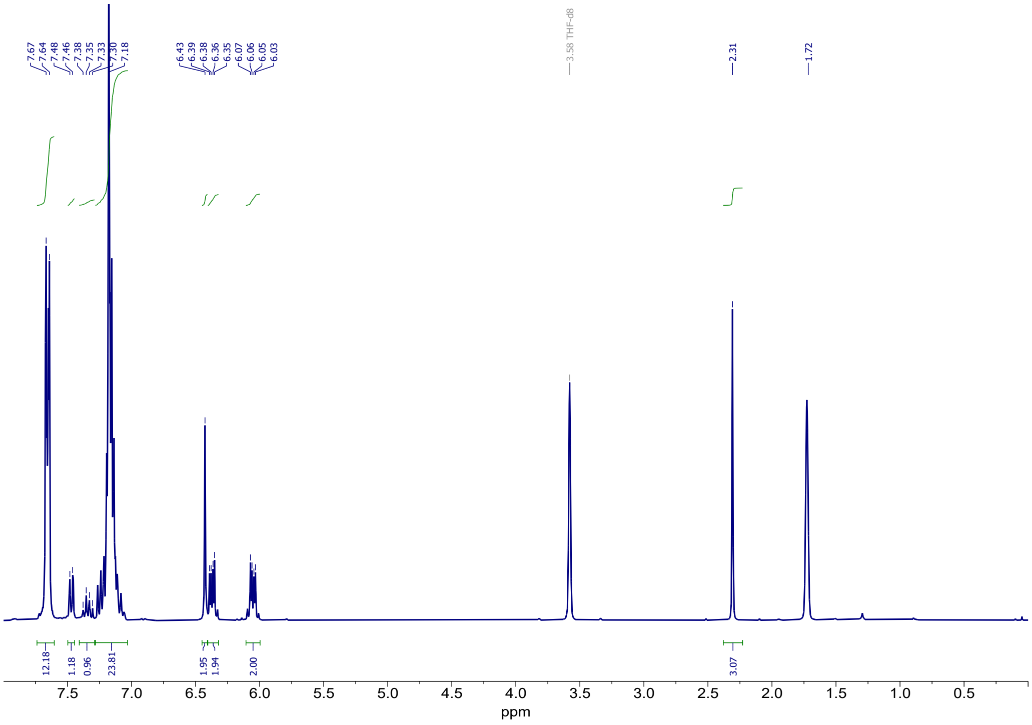

Figure S2. ^{1}H NMR spectrum (600 MHz) of K_2COT^{big} in THF-d_8.

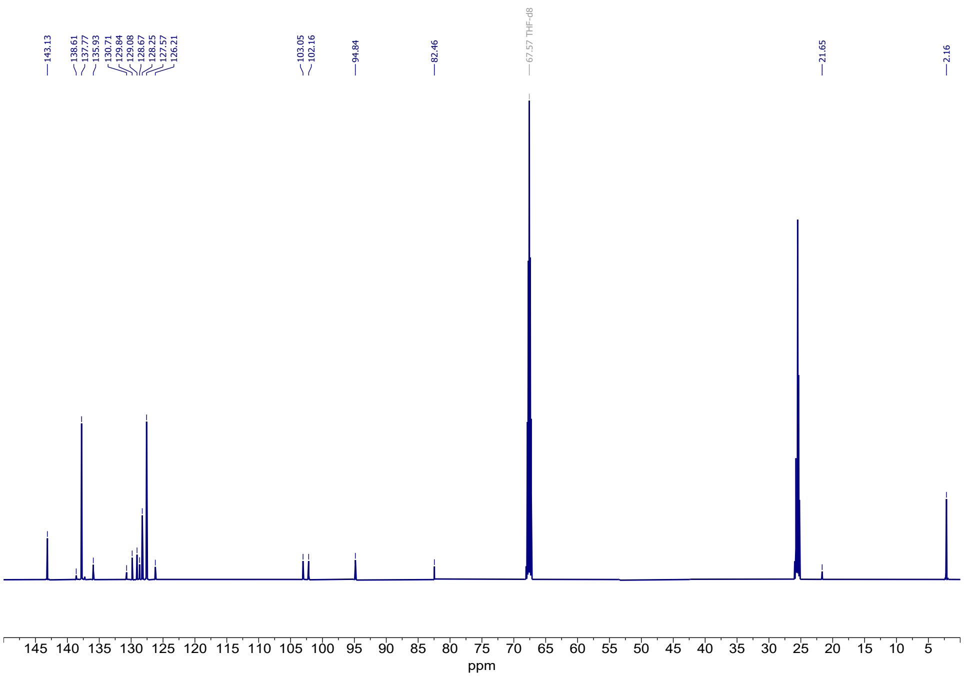

Figure S3. ^{13}C NMR spectrum (600 MHz) of K_2COT^{big} in THF-d_8.

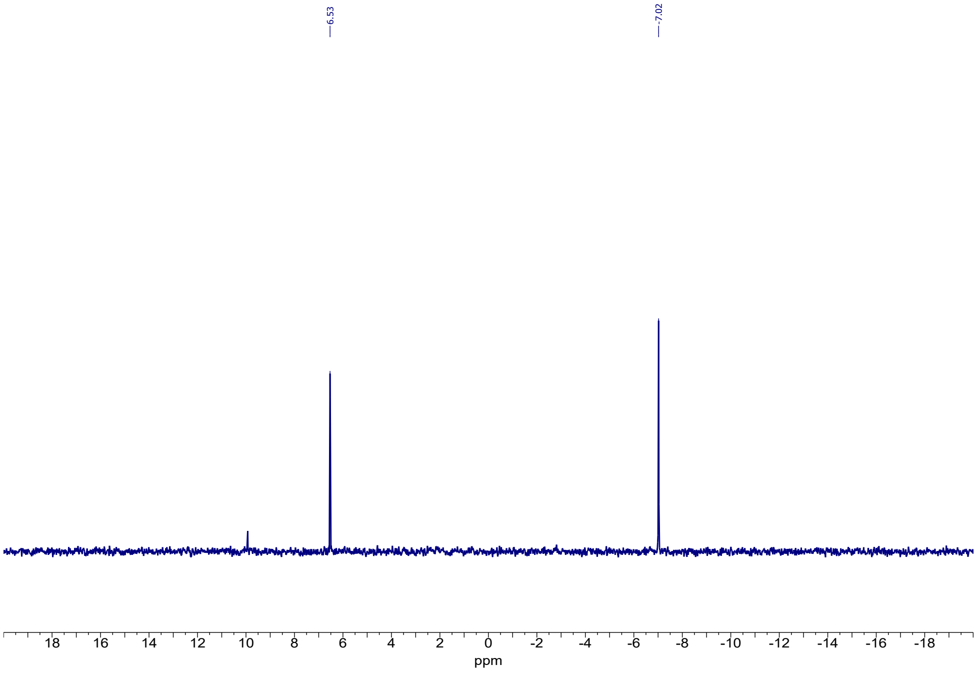

Figure S4. ^{29}Si NMR spectrum (600 MHz) of K_2COT^{Big} in THF-d_8. Hexamethyldisiloxane reference (6.53 ppm).

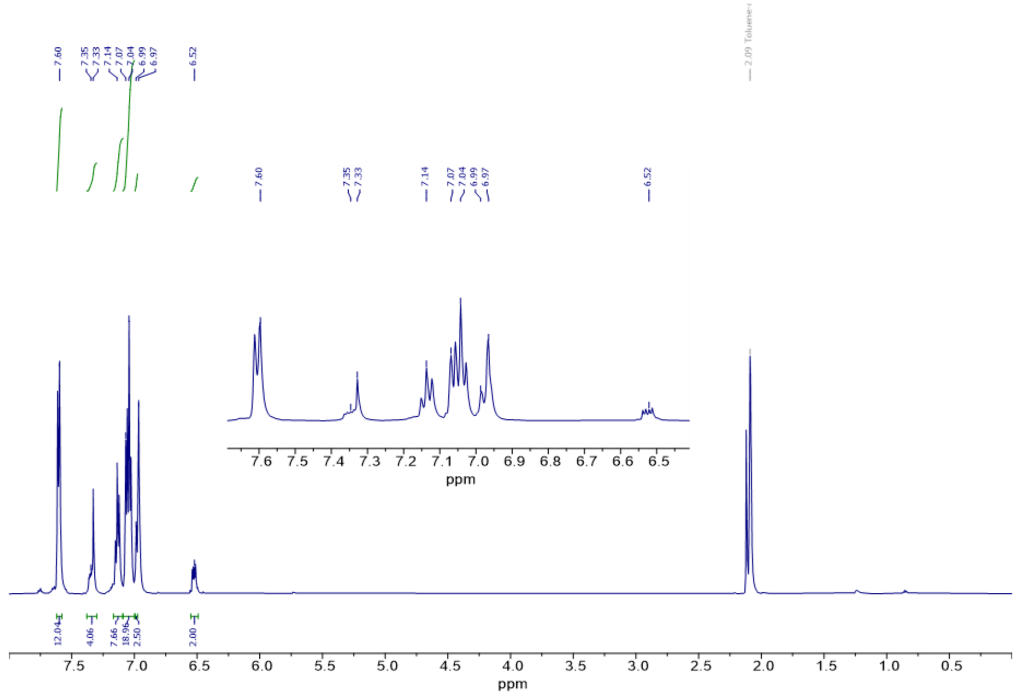

Figure S5. ^{1}H NMR spectrum (300 MHz) of **1Th** in toluene-d_8 at 350 K.

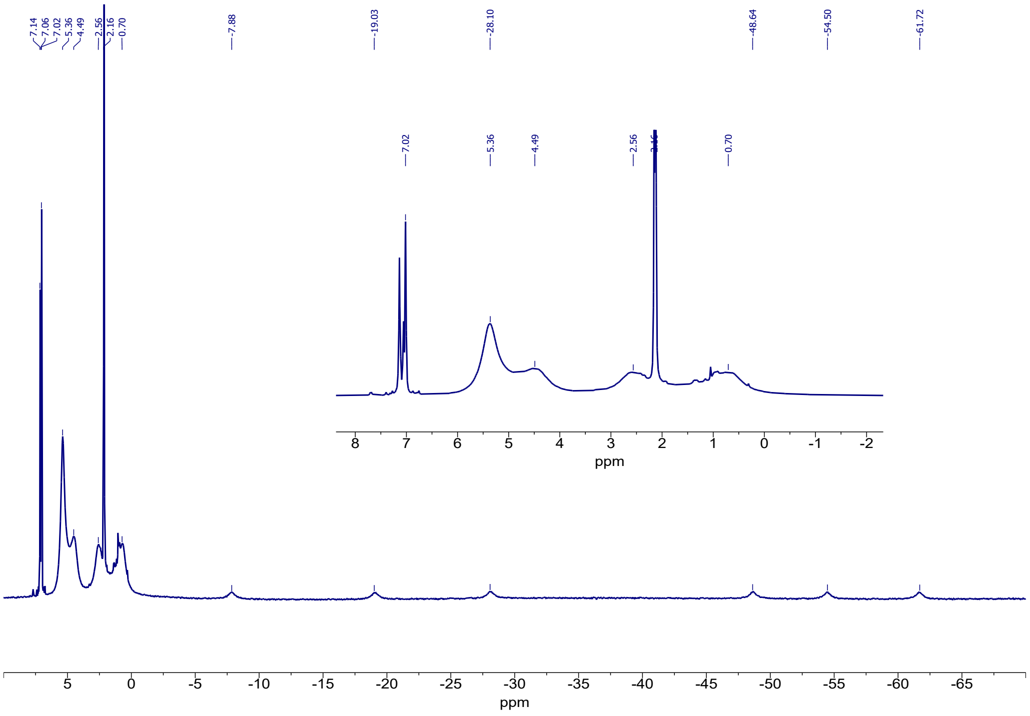

Figure S6. ^{1}H NMR spectrum (300 MHz) of **1U** in toluene-d_8 at 298 K.

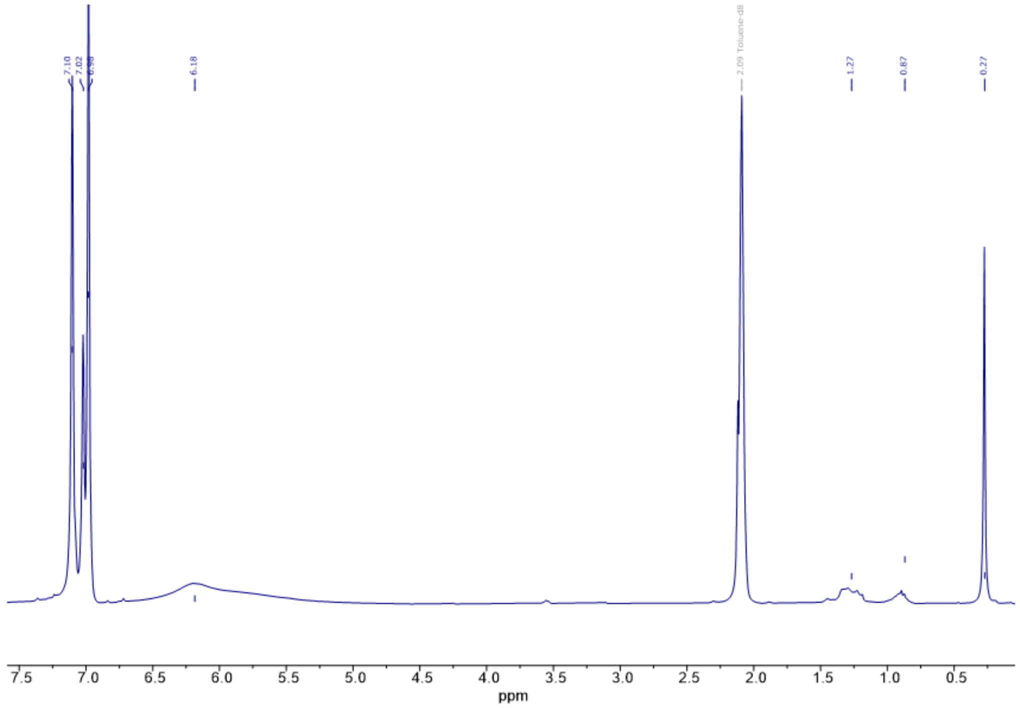

Figure S7. ^{1}H NMR spectrum (300 MHz) of **1Np** in toluene-d$_8$.

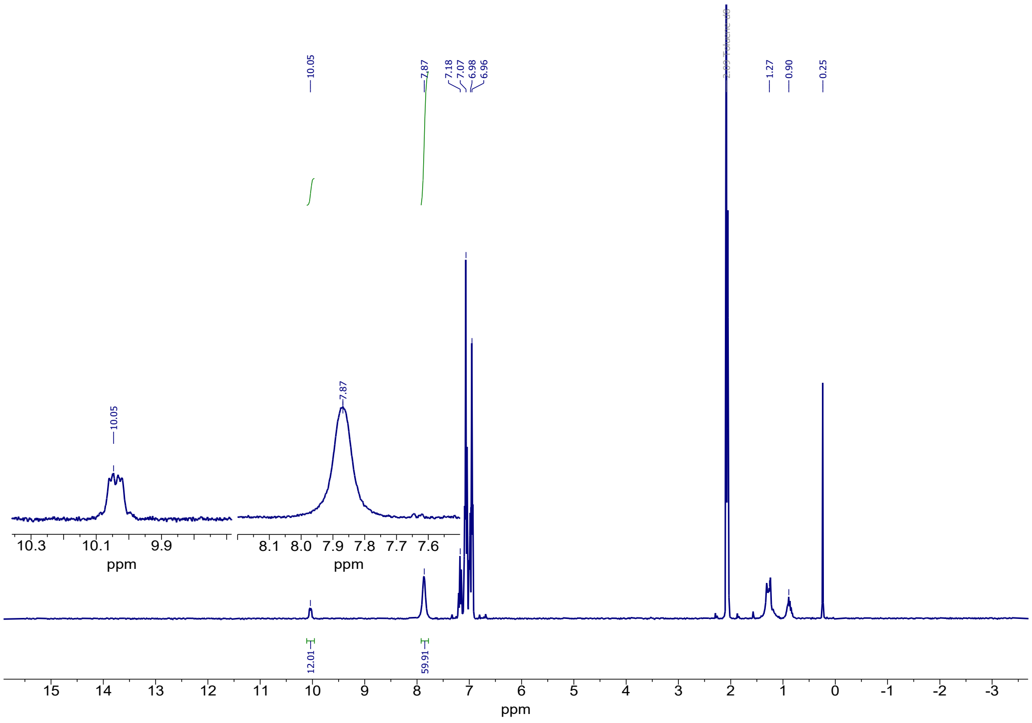

Figure S8. ^{1}H NMR spectrum (300 MHz) of **1Pu** in toluene-d$_8$.

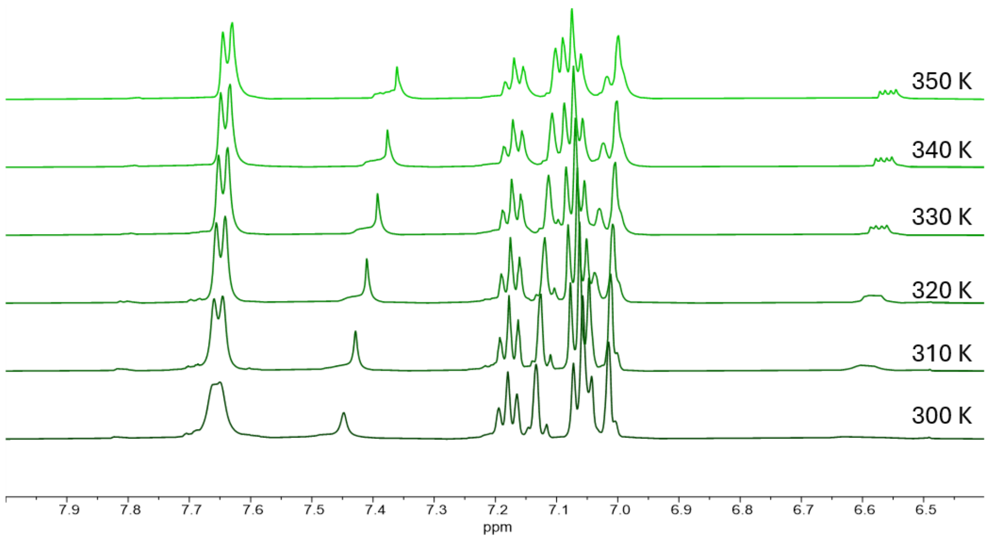

Figure S9. Stacked variable temperature ^{1}H NMR spectra of **1Th** in toluene-d_8. The temperature ranges from 300 K (lower, dark green) to 350 K (upper, light green).

S3. Crystallographic Data

Suitable crystals were selected and then mounted on an Rigaku XtaLAB Synergy-S diffractometer fitted with a HyPix-6000E detector using microfocus Cu-Kα sealed tube source. Frames were collected at 100 K or 240 K (Oxford System Cryostream) and integrated using Rigaku CrysAlys PRO software. Analytical numerical absorption corrections were performed by gaussian integration over a multifaced crystal model. The initial structure was solved by intrinsic phasing methods using the SHELXT[1] program within Olex2[2] and refined on the basis of F^2 for all unique data using the SHELXL[3] version 5 series of programs. The crystallographic information file for all structures can be found in the Cambridge Crystallographic Database Center under deposition numbers **2408395-2408403**.

Multiple structures of **1U** were collected at various temperatures (100 K & 240 K; 140 K previously reported[4]) and with different lattice solvents (benzene, **1U·bz**, and toluene, **1U·tol**) to determine if comparisons can be appropriately made across structures. In agreement with the previously reported **1U·tol** structure collected at 140 K, the **1U·tol** structure collected at 100 K crystallizes in the *C2/c* space group and display only slight expansion of unit cell parameters upon increasing temperature. At 240 K, **1U·tol** is best solved using the $C222_1$ space group. Attempted solutions within the *C2/c* space group led to worse refinement values, but nearly identical unit cell parameters. While the lattice toluene solvate was resolvable in the 100 K and 140 K collection, it could not be resolved in the 240 K collection and thus, a solvent mask ($50e^-$, 282 $Å^3$) corresponding with one toluene molecule per cell was employed.

The cell volume expands minimally between the 100K and 140K collections (7986.45(11) to 8012.1(9) $Å^3$, respectively), but then contracts sizably for the 240K collection (7820.19(11) $Å^3$), potentially indicative of a phase transformation. Notably, the An-COT_{cent} distances do not trend proportionately to the temperature (1.943(3), 1.9376(8), 1.943(2) Å for the 100 K, 140 K, and 240 K collections, respectively). Thus, we believe that it is important to compare the isostructural series based upon their respective solid-state structures collected at 240 K.

The structure of **1U·bz** was collected at 100 K and solved in the *C2/c* space group, containing one lattice solvent molecule per unit cell. The benzene solvate is disordered over the same position in an approximate 60:40 ratio. The solid-state structure of **1U·bz** was also collected at 240 K and solved in the *C2/c* space group. From 100 K to 240 K, the **1U·bz** lattice parameters expand minimally. In the 240 K **1U·bz** collection the lattice benzene is resolvable, thus **1U·bz** was used for comparison with other structures.

For completeness, the structure of **1Th·bz** was collected at both 100 K and 240 K. Both structures are solved in the *C2/c* space group with minimally expanding lattice parameters from 100 K to 240 K. Both structures contain one benzene solvate per unit cell which is disordered over the same position. The corresponding toluene solvate, **1Th·tol**, was studied at both 100 K and 240 K. At 240 K the structure was solved in the *C2/c* space group with one poorly resolved molecule of lattice toluene disordered over multiple positions, which proved to not be modellable and was treated with a solvent mask ($99e^-$, 324 $Å^3$). At 100 K the ligand framework was found to be heavily disordered making structure solution impossible.

The structure of **1Np·tol** was collected at 240 K and solved in the *C2/c* space group. Electron density corresponding to a lattice THF solvent was visible but could not be modeled with more than 25% occupancy, likely due to significant disorder. Thus, a solvent mask was used ($79e^-$, 354 $Å^3$), corresponding to one THF per asymmetric unit (or two THF per unit cell). The structure of **1Pu·bz** was likewise collected at 240 K and solved in the *C2/c* space group. The unit cell contains one lattice benzene, which was disordered similarly to the corresponding Th, and U structures.

Table S1. Crystallographic information for 100 K crystallographic data of K_2COT^{big}, **1Th**, and **1U**.

Identification code	K2_COTBig_100	Th_bz_100	U_bz_100
Empirical formula	$C_{50.75}H_{45.51}K_2Si_2$	$C_{100}H_{84}Si_4Th$	$C_{100}H_{84}Si_4U$
Formula weight	789.75	1630.07	1636.06
Temperature/K	100.00(10)	99.98(13)	100.00(10)
Crystal system	triclinic	monoclinic	monoclinic
Space group	*P-1*	*C2/c*	*C2/c*
a/Å	9.67330(10)	11.61950(10)	11.48567(5)
b/Å	13.5310(2)	26.1702(3)	26.21700(15)
c/Å	17.0094(2)	26.0886(2)	26.07195(14)
α/°	102.8950(10)	90	90
β/°	104.6870(10)	93.2440(10)	93.2186(4)
γ/°	94.3120(10)	90	90
Volume/Å^3	2078.69(5)	7920.43(13)	7838.40(7)
Z	2	4	4
ρ_{calc}g/cm^3	1.262	1.367	1.386
μ/mm^{-1}	2.822	7.016	6.783
F(000)	832.0	3320.0	3328.0
Radiation	Cu Kα (λ = 1.54184)	Cu Kα (λ = 1.54184)	Cu Kα (λ = 1.54184)
2Θ range (°)	5.55 to 159.938	6.756 to 159.932	6.744 to 159.988
Index ranges	$-12 \leq h \leq 12$, $-17 \leq k \leq 17$, $-21 \leq l \leq 18$	$-9 \leq h \leq 14$, $-33 \leq k \leq 33$, $-33 \leq l \leq 32$	$-14 \leq h \leq 11$, $-33 \leq k \leq 33$, $-33 \leq l \leq 33$
Reflections collected	46764	43944	86455
Independent reflections	8994 [R_{int} = 0.0417, R_{sigma} = 0.0325]	8476 [R_{int} = 0.0429} [R_{sigma} = 0.0323]	8523 [R_{int} = 0.0444] [R_{sigma} = 0.0207]
Data/restraints/parameters	8994/179/633	8476/477/639	8523/253/615
Goodness-of-fit on F^2	1.108	1.143	1.117
Final R indexes [I>=2σ (I)]	R_1 = 0.0357, wR_2 = 0.0978	R_1 = 0.0298, wR_2 = 0.0750	R_1 = 0.0327, wR_2 = 0.0746
Final R indexes [all data]	R_1 = 0.0386, wR_2 = 0.0998	R_1 = 0.0317, wR_2 = 0.0760	R_1 = 0.0333, wR_2 = 0.0749
Largest diff. peak/hole (e Å^{-3})	0.34/-0.37	1.34/-2.01	0.89/-1.87

Table S2. Crystallographic information for 240 K crystallographic data of **1Th** and **1U**

Identification code	Th_bz_240	U_bz_240	U_tol_240
Empirical formula	$C_{100}H_{84}Si_4Th$	$C_{100}H_{84}Si_4U$	$C_{88}H_{72}Si_4U$
Formula weight	1630.07	1636.06	1479.84
Temperature/K	240.0(2)	240.0(3)	239.99(10)
Crystal system	monoclinic	monoclinic	orthorhombic
Space group	*C2/c*	*C2/c*	$C222_1$
a/Å	11.66450(10)	11.55920(10)	11.57920(10)
b/Å	26.2205(2)	26.3815(2)	25.8345(2)
c/Å	26.4867(2)	26.4452(2)	26.1420(2)
α/°	90	90	90
β/°	93.0410(10)	93.1470(10)	90
γ/°	90	90	90
Volume/Å^3	8089.52(11)	8052.28(11)	7820.19(11)
Z	4	4	4
ρ_{calc}g/cm^3	1.338	1.350	1.257
μ/mm^{-1}	6.869	6.603	6.743
F(000)	3320.0	3328.0	2992.0
Radiation	Cu Kα (λ = 1.54184)	Cu Kα (λ = 1.54184)	Cu Kα (λ = 1.54184)
2Θ range for data collection/°	6.684 to 159.944	6.694 to 159.57	6.762 to 160.054
Index ranges	-14 ≤ h ≤ 10, -33 ≤ k ≤ 33, -33 ≤ l ≤ 33	-14 ≤ h ≤ 14, -33 ≤ k ≤ 33, -33 ≤ l ≤ 28	-14 ≤ h ≤ 13, -31 ≤ k ≤ 32, -33 ≤ l ≤ 33
Reflections collected	46213	46098	88075
Independent reflections	8639 [R_{int} = 0.0544, R_{sigma} = 0.0430]	8629 [R_{int} = 0.0431, R_{sigma} = 0.0318]	8492 [R_{int} = 0.0460, R_{sigma} = 0.0217]
Data/restraints/parameters	8639/384/639	8629/285/560	8492/12/420
Goodness-of-fit on F^2	1.103	1.112	1.053
Final R indexes [I>=2σ (I)]	R_1 = 0.0290, wR_2 = 0.0757	R_1 = 0.0289, wR_2 = 0.0761	R_1 = 0.0358, wR_2 = 0.0879
Final R indexes [all data]	R_1 = 0.0304, wR_2 = 0.0766	R_1 = 0.0301, wR_2 = 0.0767	R_1 = 0.0364, wR_2 = 0.0882
Largest diff. peak/hole / e Å^{-3}	0.77/-1.13	1.22/-0.91	1.00/-1.28

Table S3. Crystallographic information for 240 K crystallographic data of **1Np** and **1Pu**.

Identification code	Np_tol_240K	Pu_bz_240
Empirical formula	$C_{96}H_{88}NpO_2Si_4$	$C_{100}H_{84}PuSi_4$
Formula weight	1623.02	1640.03
Temperature(K)	239.99(10)	239.99(10)
Crystal system	monoclinic	monoclinic
Space group	*C2/c*	*C2/c*
a/Å	11.5922(2)	11.5499(2)
b/Å	26.7070(4)	26.4310(7)
c/Å	26.0296(7)	26.5815(7)
α/°	90	90
β/°	91.503(2)	93.038(2)
γ/°	90	90
Volume (Å^3)	8055.8(3)	8103.3(3)
Z	4	4
ρ_{calc}g/cm^3	1.338	1.344
μ/mm^{-1}	7.159	6.611
F(000)	3316.0	3336.0
Radiation	Cu Kα (λ = 1.54184)	Cu Kα (λ = 1.54184)
2Θ range (°)	6.794 to 136.492	6.66 to 144.204
Index ranges	-13 ≤ h ≤ 13, -31 ≤ k ≤ 24, -30 ≤ l ≤ 31	-14 ≤ h ≤ 14, -28 ≤ k ≤ 32, -29 ≤ l ≤ 32
Reflections collected	41036	42845
Independent reflections	7180 [R_{int} = 0.0740, R_{sigma} = 0.0429]	7882 [R_{int} = 0.0513, R_{sigma} = 0.0329]
Data/restraints/parameters	7180/48/433	7882/232/529
Goodness-of-fit on F^2	1.191	1.038
Final R indexes [I>=2σ (I)]	R_1 = 0.0593, wR_2 = 0.1458	R_1 = 0.0309, wR_2 = 0.0741
Final R indexes [all data]	R_1 = 0.0651, wR_2 = 0.1484	R_1 = 0.0392, wR_2 = 0.0772
Largest diff. peak/hole / e Å^{-3}	2.85/-2.58	0.59/-1.40

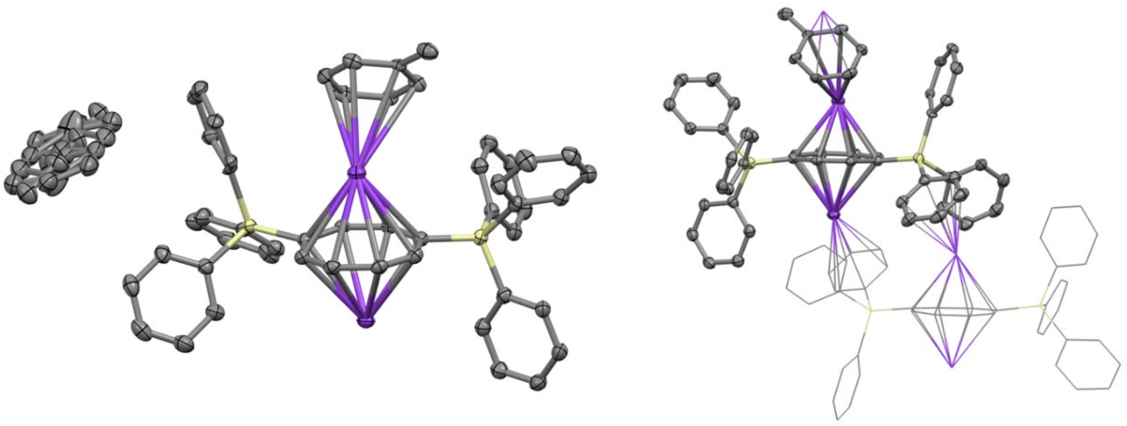

Figure S10. Molecular structure of K_2COT^{big} with thermal ellipsoids at 50%, (left) showing asymmetric unit and right showing adjacent positions in wireframe. Hydrogen atoms, minor component of disordered phenyl ring and one component of bridging toluene omitted for clarity. Solvent position on left is disordered toluene/*n*-hexane at approximately 1:1 ratio, with toluene additionally disordered over an inversion center. Grey: C, purple: K, cream: Si.

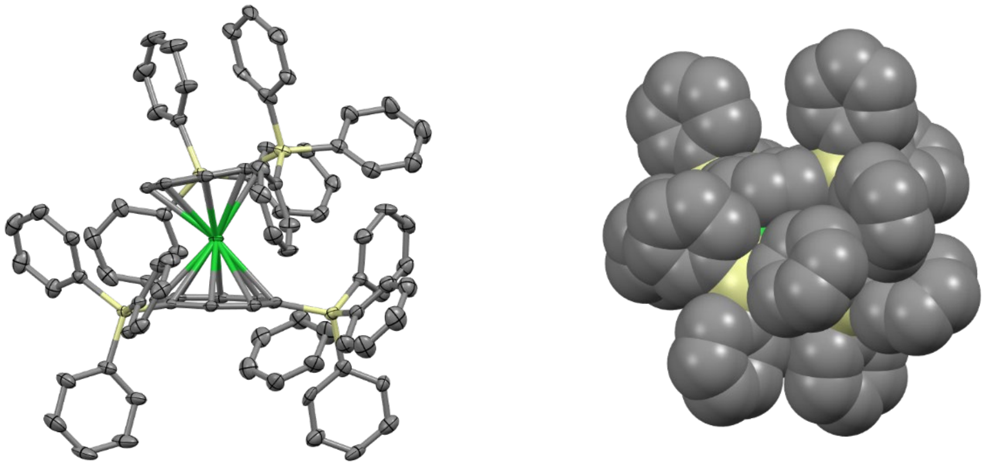

Figure S11. Left: molecular structure of **1Th·bz** collected at 100 K with thermal ellipsoids at 50%. Hydrogen atoms and minor component of disordered phenyl rings and benzene solvate omitted for clarity. Right: space filling model of **1Th·bz** in the same orientation. Light Green: Th, grey: C, cream: Si.

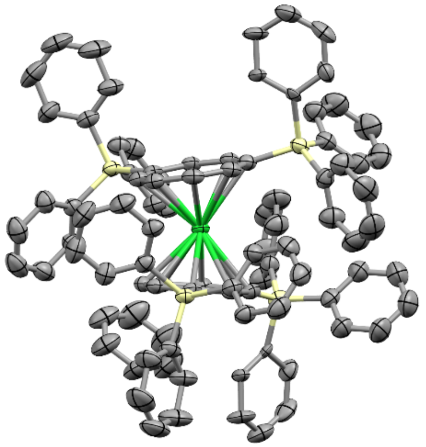

Figure S12. Molecular structure of **1Th·bz** collected at 240 K with thermal ellipsoids at 50%. Hydrogen atoms, minor component of disordered phenyl rings and benzene solvate omitted for clarity. Light green: Th, grey: C, cream: Si.

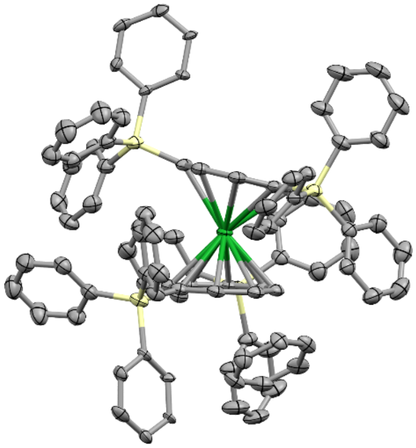

Figure S13. Molecular structure of **1U·bz** collected at 100 K with thermal ellipsoids at 50%. Hydrogen atoms, minor component of disordered phenyl rings and benzene solvate omitted for clarity. Green: U, grey: C, cream: Si.

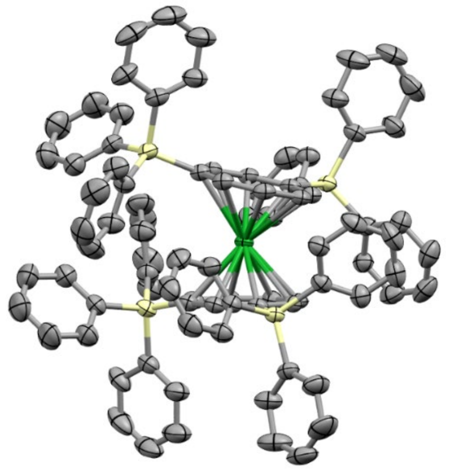

Figure S14. Molecular structure of **1U·bz** collected at 240 K with thermal ellipsoids at 50%. Hydrogen atoms, minor component of disordered phenyl rings and benzene solvate omitted for clarity. Green: U, grey: C, cream: Si.

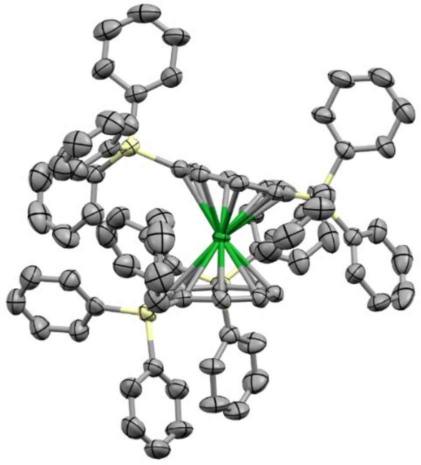

Figure S15. Molecular structure of **1U·tol** collected at 240 K with thermal ellipsoids at 50%. Hydrogen atoms omitted for clarity. Green: U, grey: C, cream: Si.

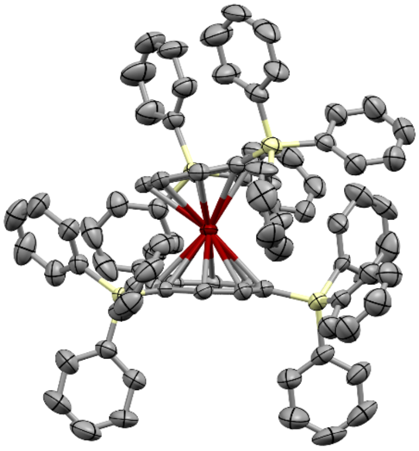

Figure S16. Molecular structure of **1Np·tol** collected at 240 K with thermal ellipsoids at 50%. Hydrogen atoms, minor component of disordered phenyl rings omitted for clarity. Red: Np, grey: C, cream: Si.

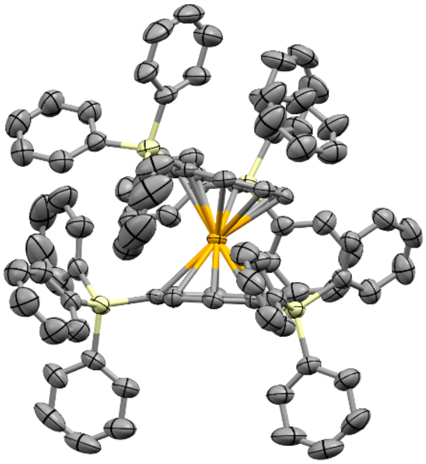

Figure S17. Molecular structure of **1Pu·bz** collected at 240 K with thermal ellipsoids at 50%. Hydrogen atoms, minor component of disordered phenyl rings and benzene solvate omitted for clarity. Orange: Pu, grey: C, cream: Si.

S4. UV-VIS Spectroscopy

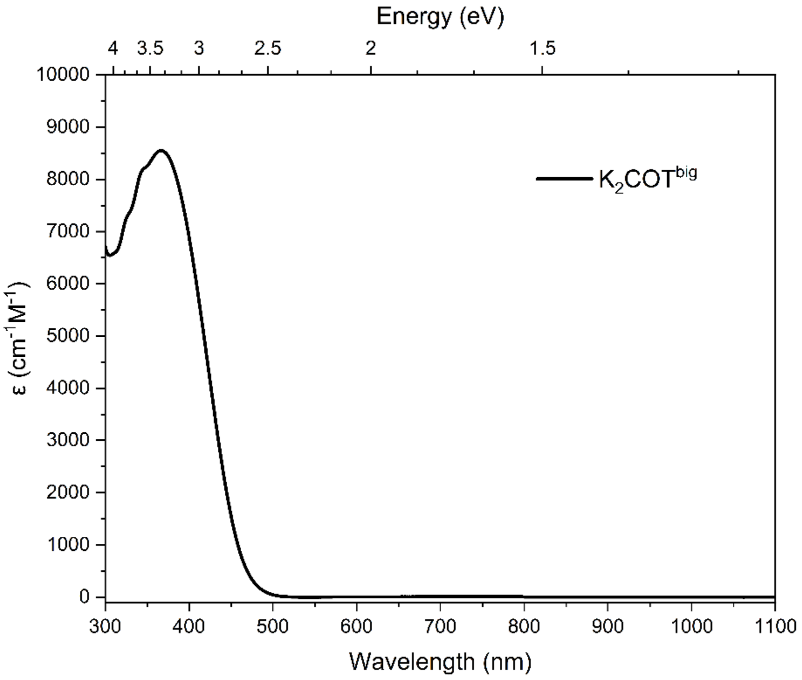

Figure S18. UV-Vis spectrum of K_2COT^{big} [50 μM] in toluene.

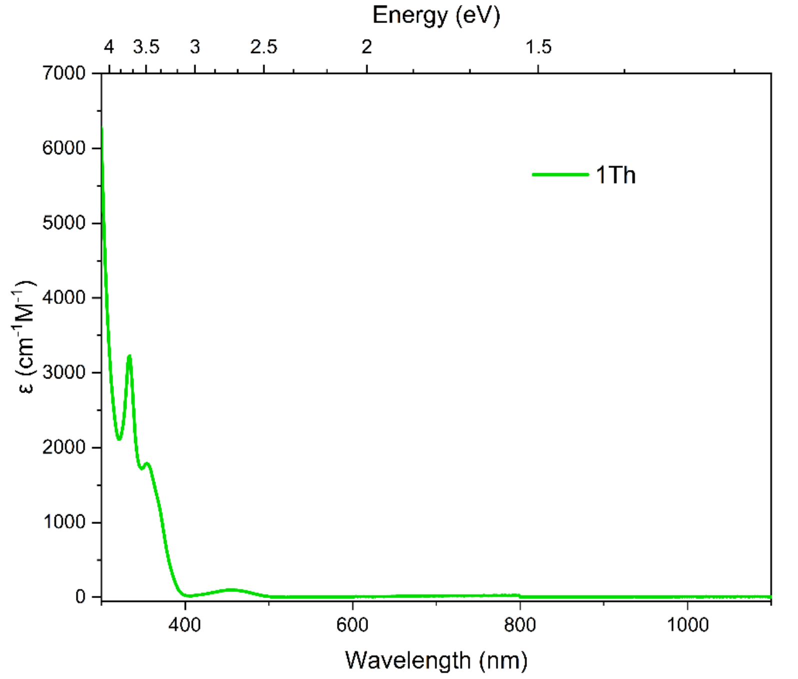

Figure S19. UV-Vis spectrum of **1Th** [50 μM] in toluene.

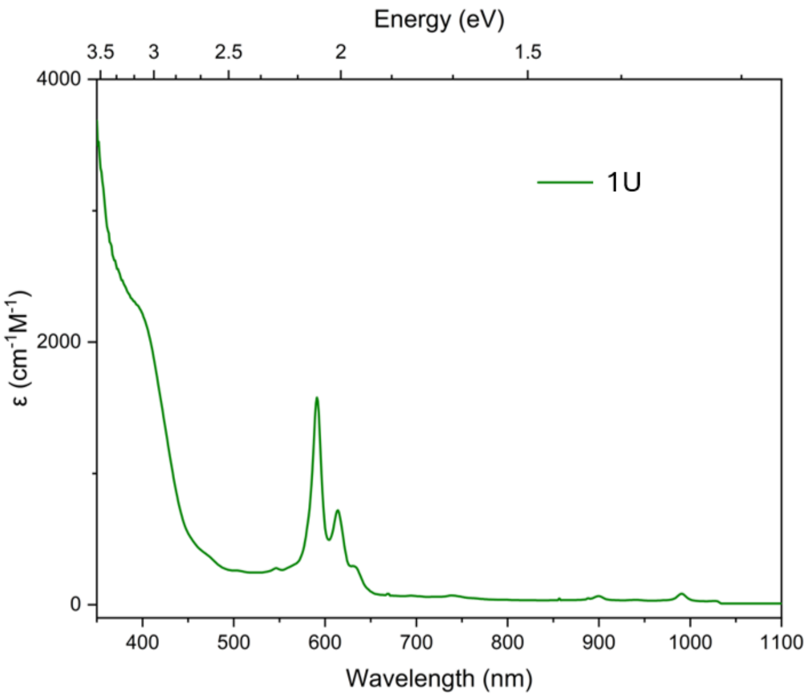

Figure S20. UV-Vis spectrum of **1U** [50 µM] in toluene.

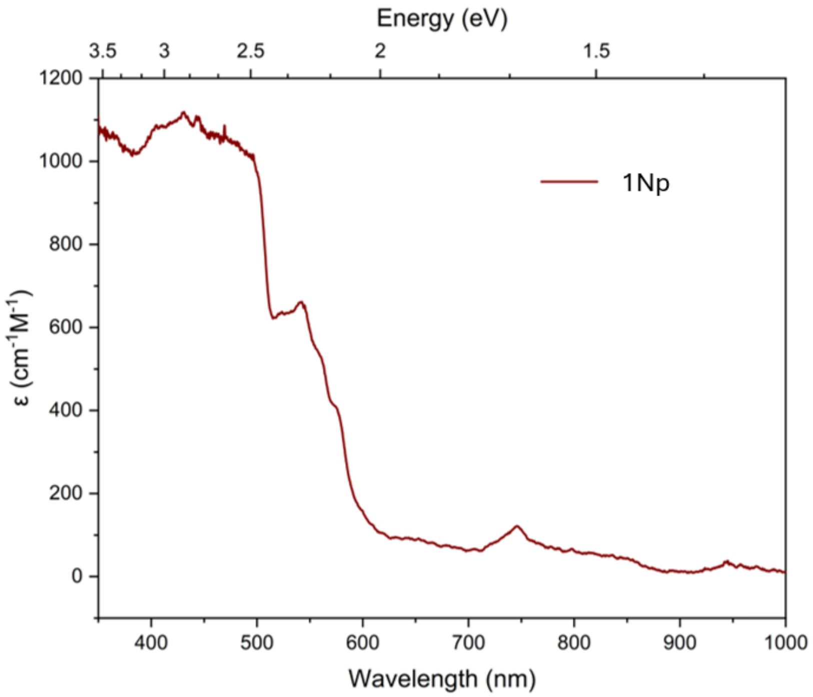

Figure S21. UV-Vis spectrum of **1Np** [1.1 mM] in toluene

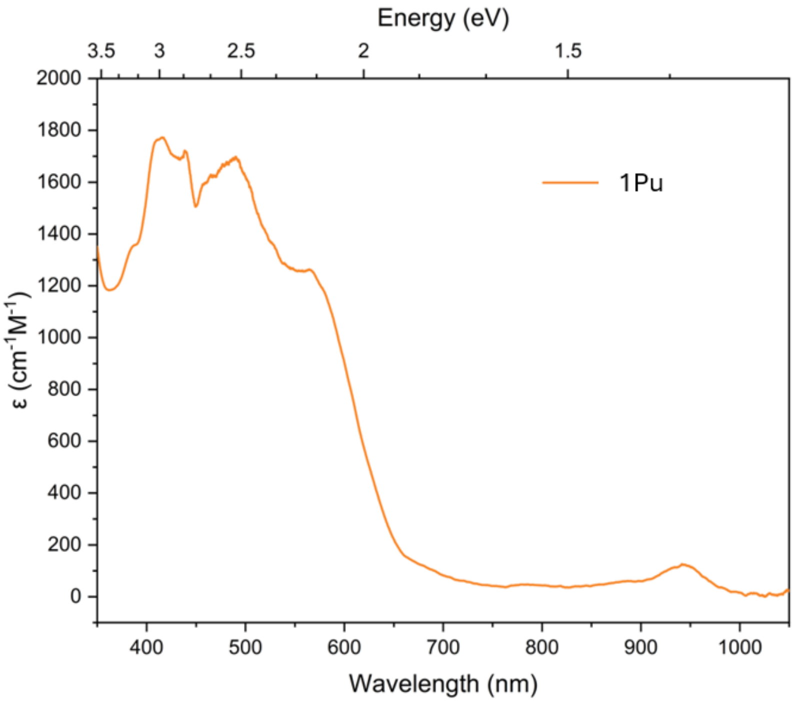

Figure S22. UV-Vis spectrum of **1Pu** [1.1mM] in toluene.

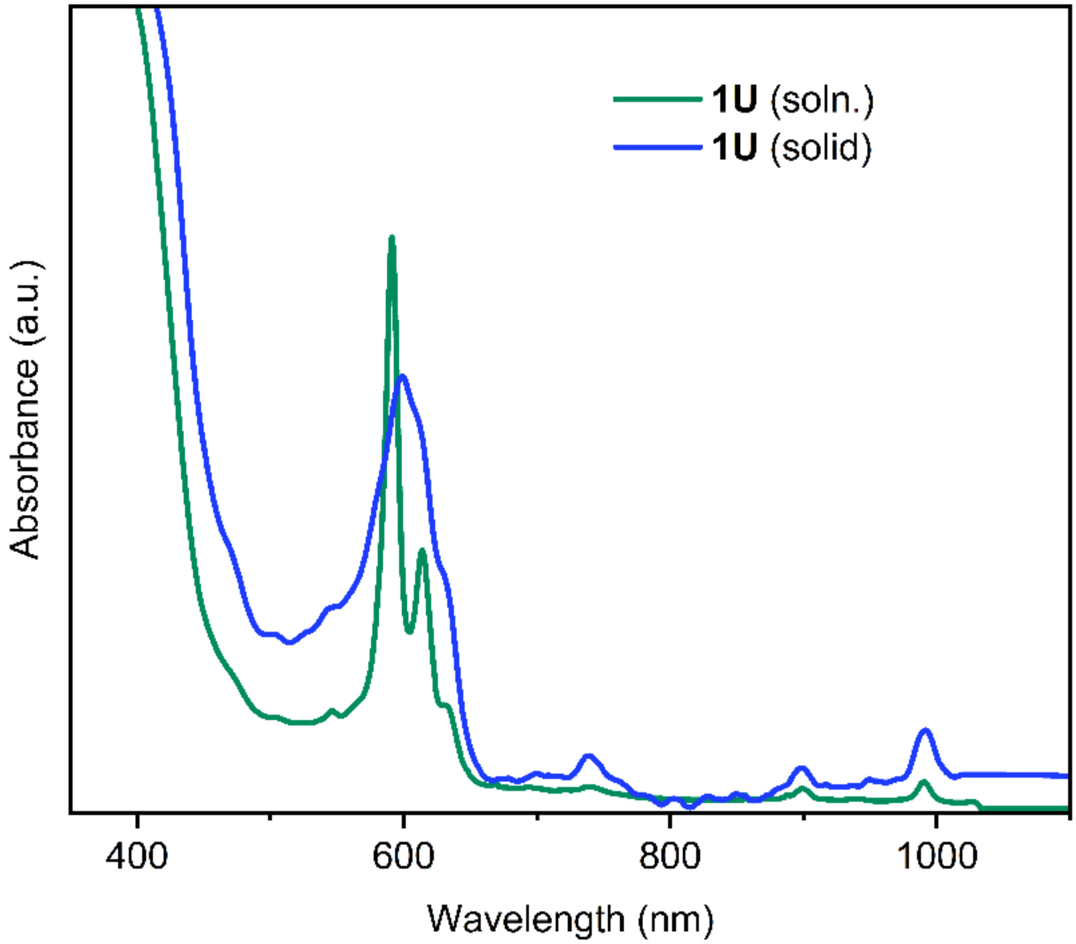

Figure S23. Normalized UV-Vis spectrum of **1U** in toluene (green) and crystalline **1U** (blue).

S5 Additional Characterization

S5.1. IR spectroscopy

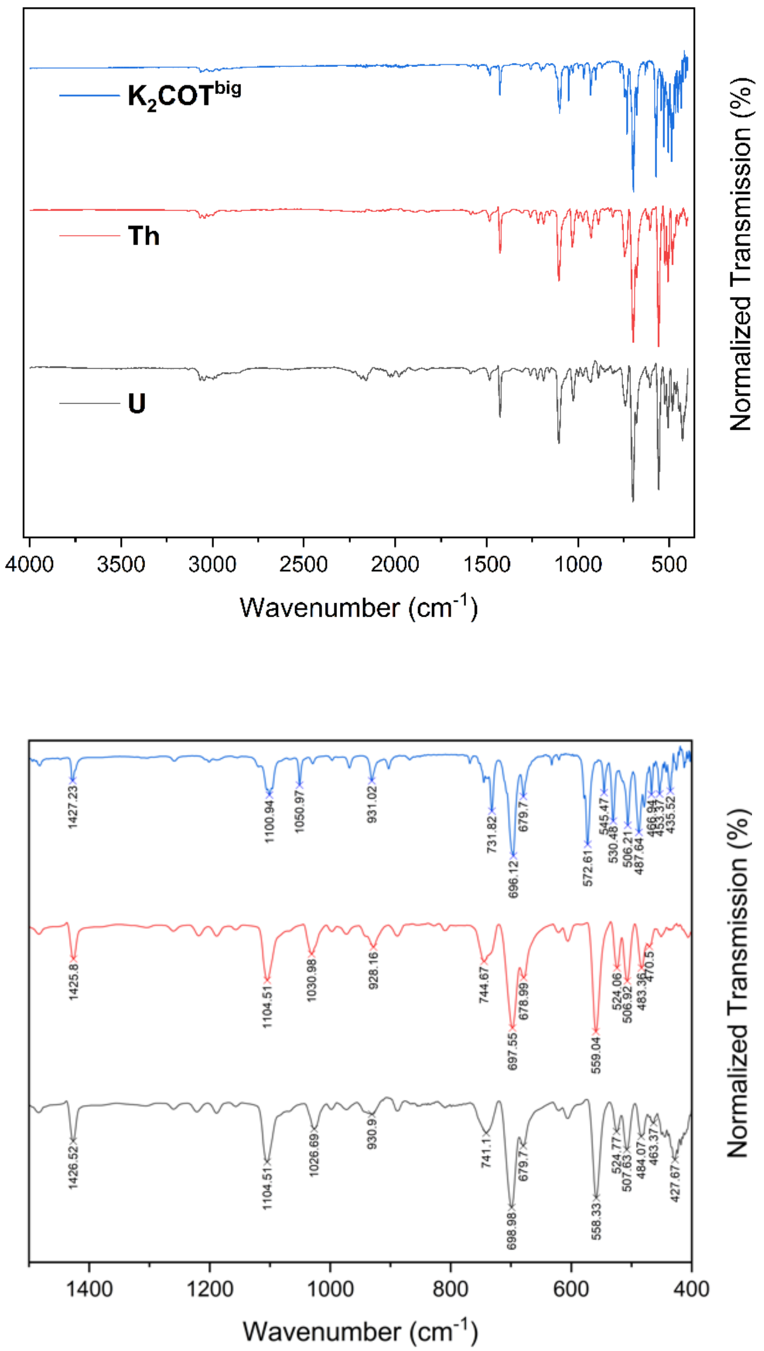

Figure S24. Upper: IR Vibrational bands for K_2COT^{big} (black), **1Th** (Red), **1U** (Blue). Lower: Zoomed spectra with labeled peaks.

S5.2 Fluorescence spectroscopy

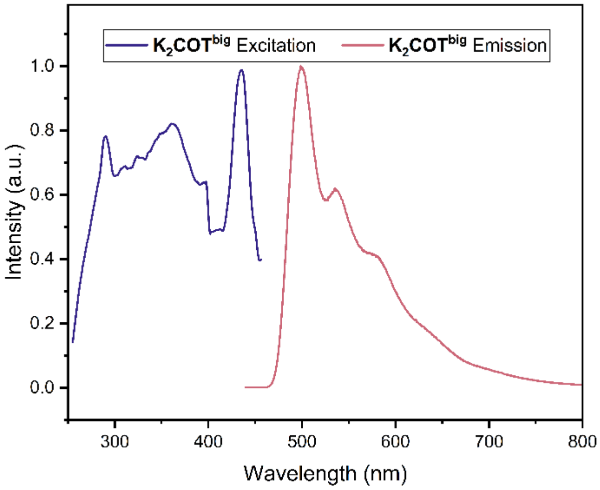

Figure S25.. Fluorescence spectra for K_2COT^{big} recorded in THF (20 µM). Excitation spectrum (purple) was measured an emission wavelength of 499 nm. Emission spectrum (red) was excited at 436 nm.

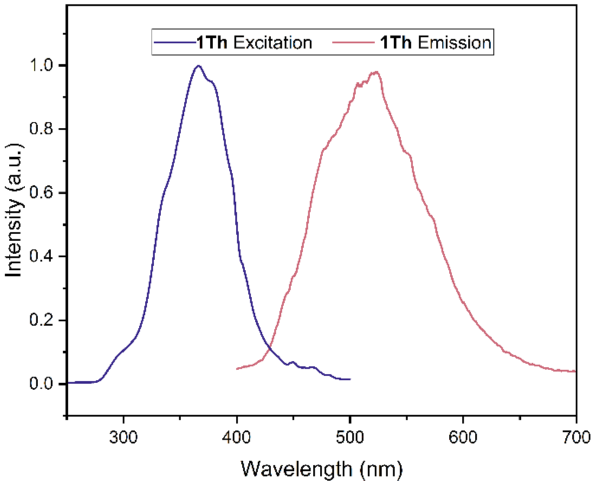

Figure S26. Fluorescence spectra for **1Th** recorded in DCM (20 µM). Excitation spectrum (purple) was measured an emission wavelength of 520 nm. Emission spectrum (red) was excited at 365 nm.

6. Computational Analysis

TableS4. PBE0 calculations. Selected bonding parameters from DFT calculated optimized geometry of **1An** (An = Th, U, Np, Pu). Bond distances in Å and angles in degrees. Data in parentheses correspond to solvent-optimized geometries (solvent model for toluene).

Compound	M-COT_{cent} distance	(COT^{big})C-C_{avg} distance	Bend angle	Twist angle
**1Th**	2.022 (2.020)	1.408 (1.409)	166.8 (166.8)	97.1 (97.8)
**1U**	1.952 (1.949)	1.406 (1.406)	167.9 (168.1)	98.1 (98.3)
**1Np**	1.947 (1.935)	1.406 (1.406)	167.6 (167.3)	97.4 (97.9)
**1Pu**	1.953 (1.932)	1.406 (1.407)	165.8 (167.7)	97.2 (98.0)

Table S5. PBE0 calculations. Kohn-Sham DFT calculated C=C parameters in **1An** (An = Th, U, Np, Pu) complexes in gas phase and solvent phase (toluene) optimized geometries. Bond distances in Å.

Bond parameter	**1Th**		**1U**		**1Np**		**1Pu**	
	gas	toluene	gas	toluene	gas	toluene	gas	toluene
C1-C2 (COT unit-I)	1.415	1.415	1.409	1.410	1.405	1.412	1.406	1.412
C2-C3 (COT unit-I)	1.405	1.405	1.403	1.404	1.408	1.401	1.410	1.406
C3-C4 (COT unit-I)	1.414	1.414	1.413	1.413	1.407	1.415	1.404	1.410
C4-C5 (COT unit-I)	1.414	1.414	1.412	1.412	1.416	1.411	1.418	1.414
C5-C6 (COT unit-I)	1.401	1.402	1.400	1.400	1.396	1.399	1.394	1.399
C6-C7 (COT unit-I)	1.402	1.402	1.399	1.399	1.404	1.402	1.406	1.402
C7-C8 (COT unit-I)	1.402	1.402	1.400	1.401	1.395	1.397	1.395	1.401
C8-C1 (COT unit-I)	1.414	1.414	1.412	1.412	1.416	1.413	1.417	1.413
C1-C2 (COT unit-II)	1.415	1.415	1.409	1.410	1.405	1.412	1.406	1.412
C2-C3 (COT unit-II)	1.405	1.405	1.403	1.404	1.408	1.401	1.410	1.406
C3-C4 (COT unit-II)	1.414	1.414	1.413	1.413	1.407	1.415	1.404	1.410
C4-C5 (COT unit-II)	1.414	1.414	1.412	1.412	1.416	1.411	1.418	1.414
C5-C6 (COT unit-II)	1.401	1.402	1.400	1.400	1.396	1.399	1.394	1.399
C6-C7 (COT unit-II)	1.402	1.402	1.399	1.399	1.404	1.402	1.406	1.402
C7-C8 (COT unit-II)	1.402	1.402	1.400	1.401	1.395	1.397	1.395	1.401
C8-C1 (COT unit-II)	1.414	1.414	1.412	1.412	1.416	1.413	1.417	1.413

Table S6. PBE0 calculations with toluene solvent model. Energies of frontier molecular orbitals (FMOs) with their energy gaps in **1An** (An = Th, U, Np, Pu) complexes. The FMOs considered here are according to their similarity with the corresponding NTOs responsible for the transition at 3.8 eV, 3.5 eV, 2.4 eV, 2.2 eV respectively. Energies are in eV.

Compound	Donor	Acceptor	ΔE (donor-acceptor)
**1Th**	-5.80	-1.28	4.52
**1U**	-6.77	-1.87	3.88
**1Np**	-5.55	-2.35	3.62
**1Pu**	-6.46	-2.71	3.51

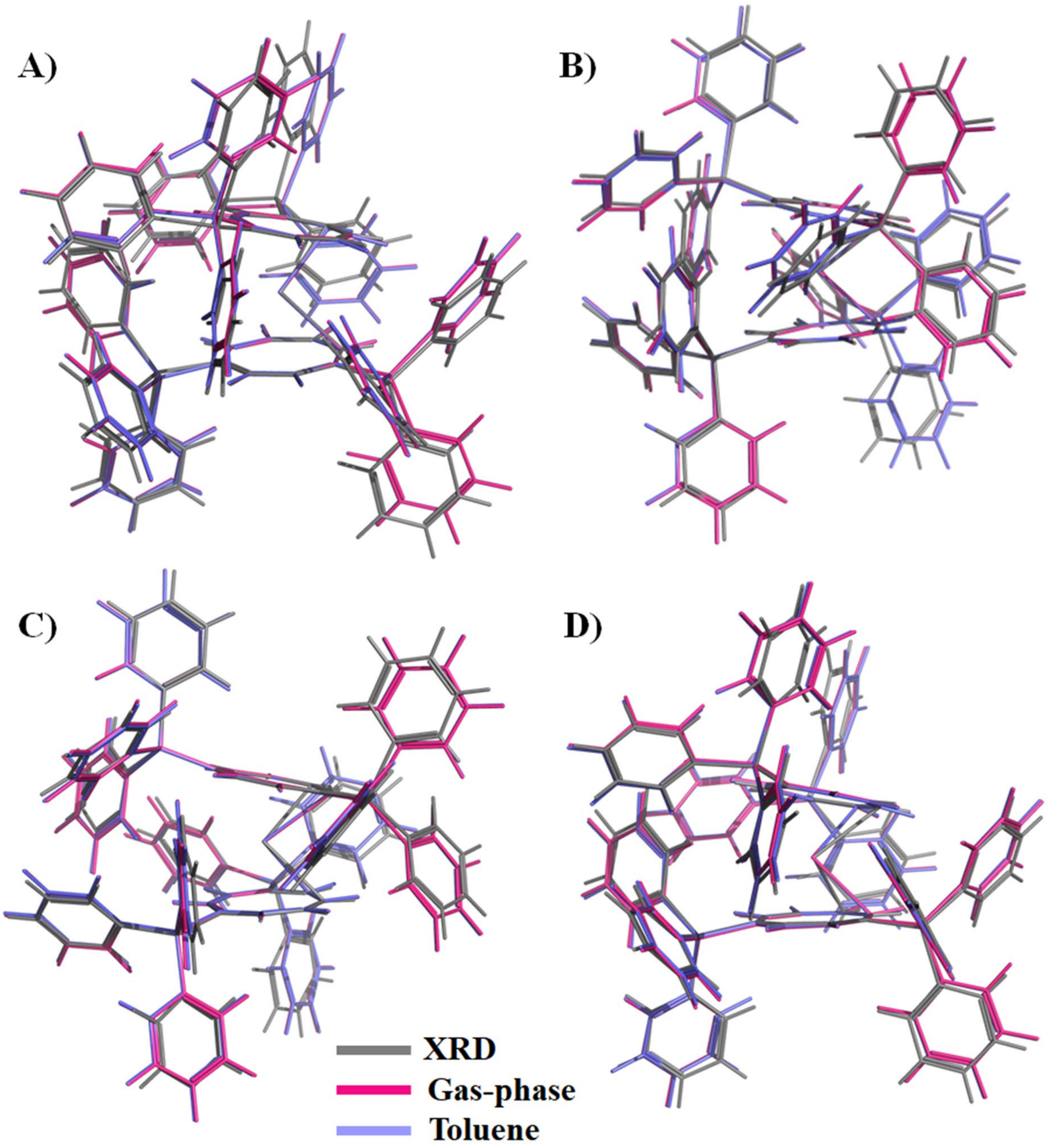

Figure S27. PBE0 calculations. Side view of the superimposition of the hydrogen-optimized (gray) X-ray crystal structure vs. gas phase (pink) vs. solvent phase (blue, in toluene) fully optimized structure of **1An** where An = Th(A), U(B), Np(C) and Pu(D).

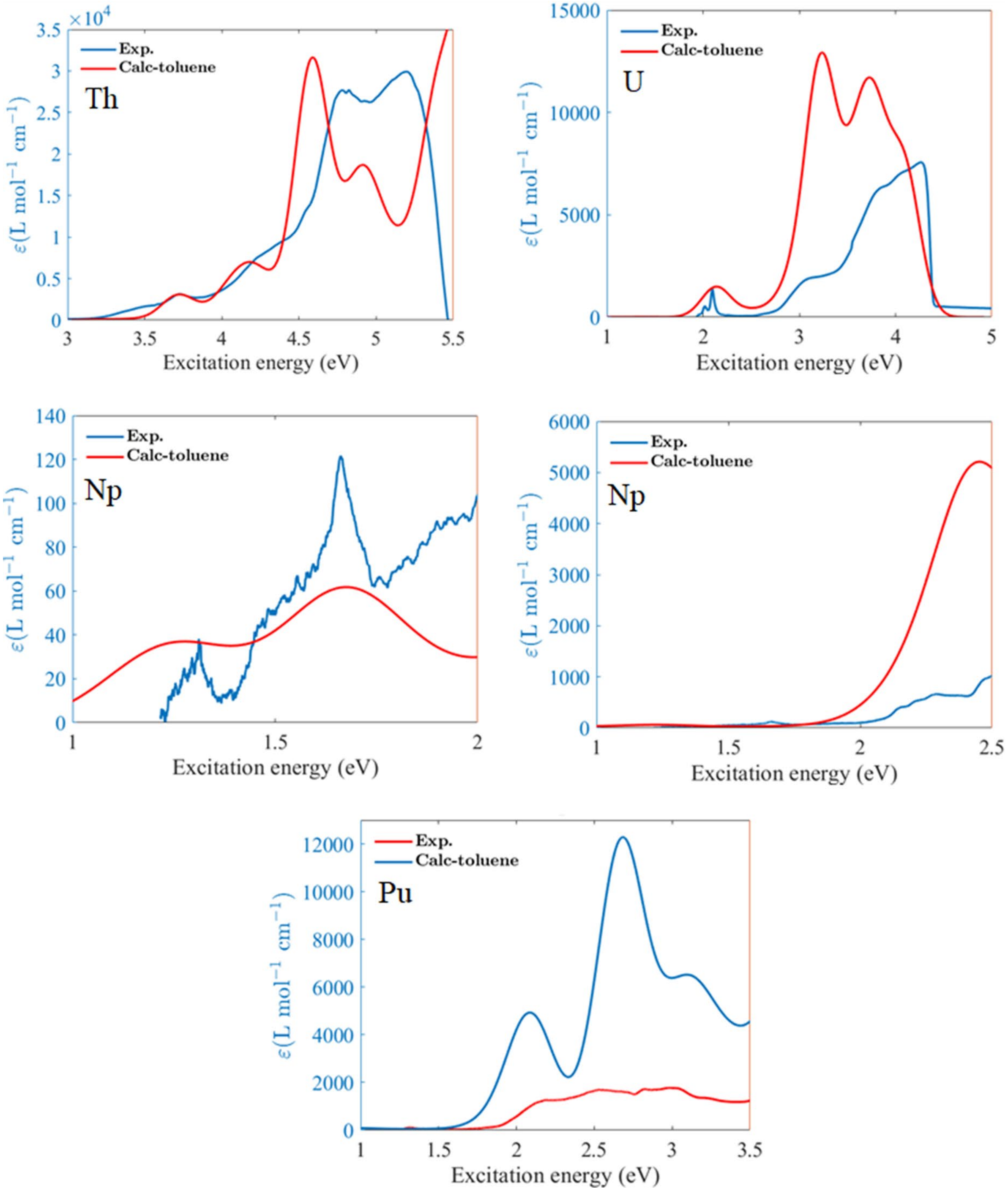

Figure S28. TPSSh calculations. Overlay of experimentally recorded (red), TDDFT computed (in toluene, blue) UV-Vis spectrum of **1An**, where An = Th (upper panel, left: blue shift 0.15 eV), U (upper panel, right: blue shift 0.15 eV), Np covering the range between 1 to 2 eV (middle panel, left: blue shift 0.15 eV), Np covering the range between 1 to 2.5 eV (middle panel, right: red shift 0.3 eV) and Pu (bottom panel: red shift 0.3 eV). The Gaussian broadening parameter for the calculated spectra was σ = 0.1 eV for Th and 0.15 eV for U-Pu complexes. The calculated spectra were red or blue shifted as noted to align the peaks.

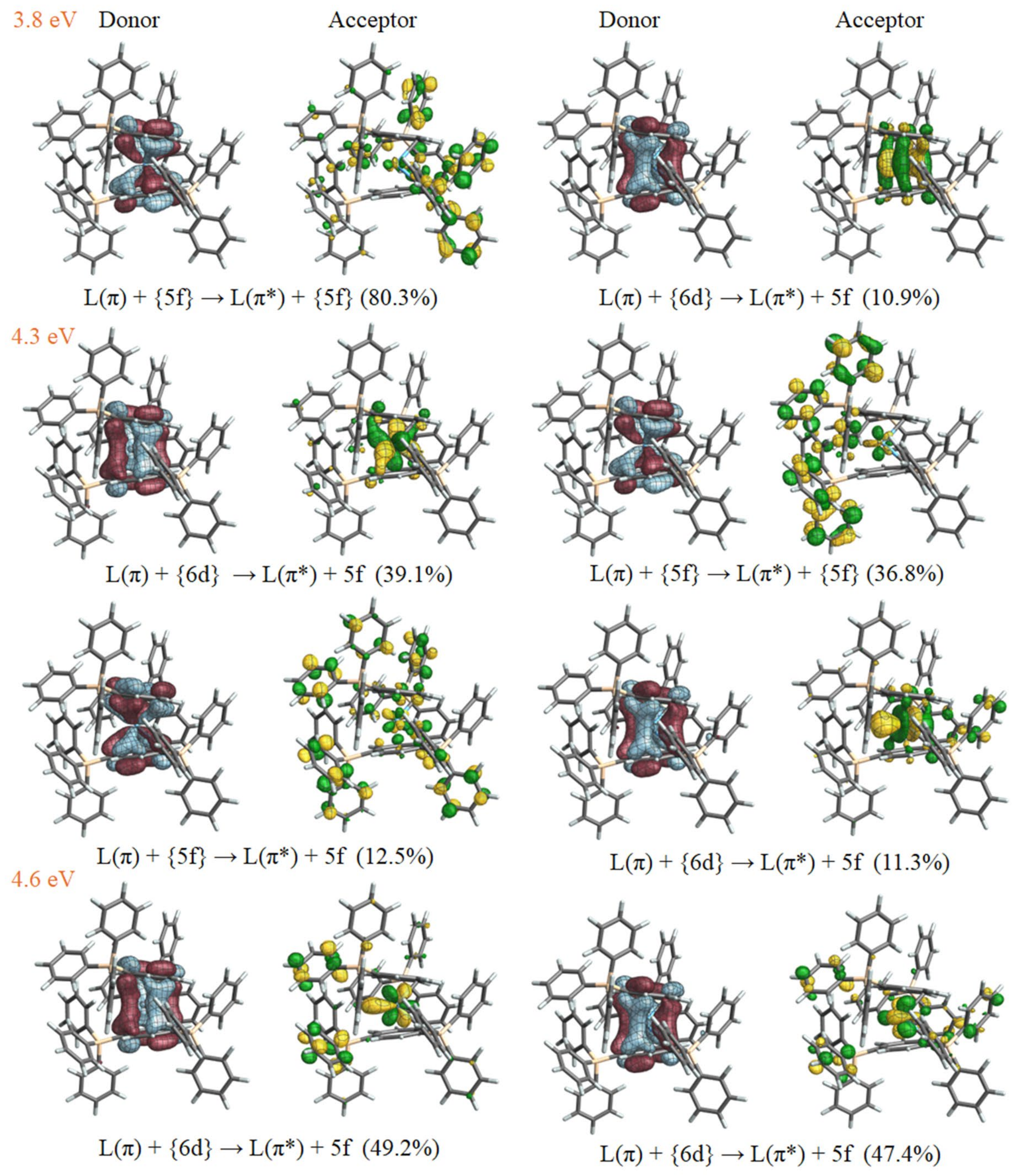

Figure S29. TPSSh calculations. Natural transition orbitals (NTOs, ±0.03 isosurfaces) corresponding to the important transitions in TD-DFT computed UV-Vis spectrum of **1Th** in toluene up to 5.0 eV. Weight-% of the NTOs corresponding to various transitions are listed. {} denotes a visible but small contribution. Transition energies given in the figure correspond to the calculations without applying a shift.

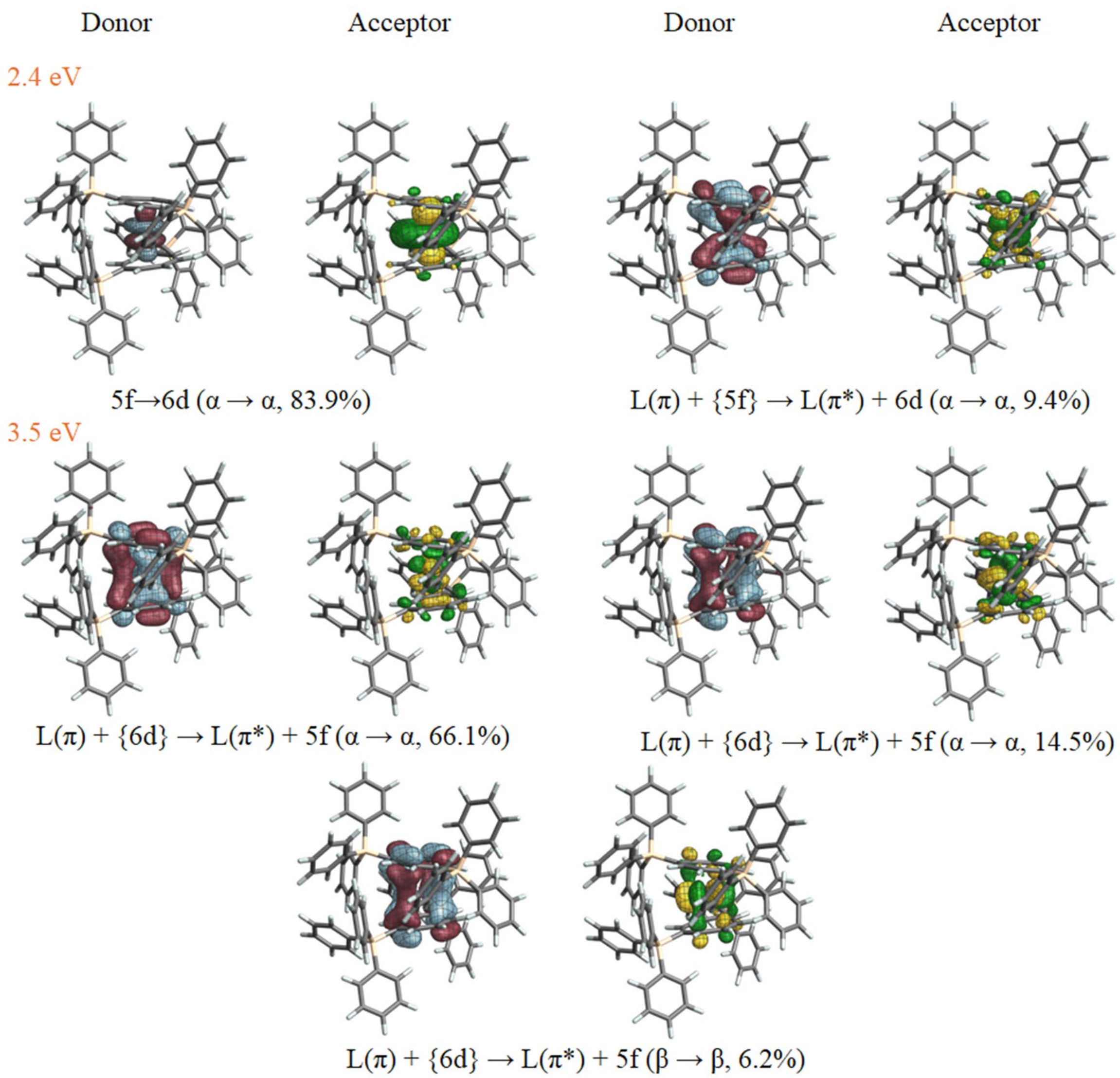

Figure S30. TPSSh calculations. Natural transition orbitals (NTOs, ±0.03 isosurfaces) corresponding to the important transitions in TD-DFT computed UV-Vis spectrum of **1U** in toluene up to 3.5 eV. Weight-% of the NTOs corresponding to α→α and β→β transitions are listed. {} denotes a visible but small contribution. Transition energies given in the figure correspond to the calculations without applying a shift.

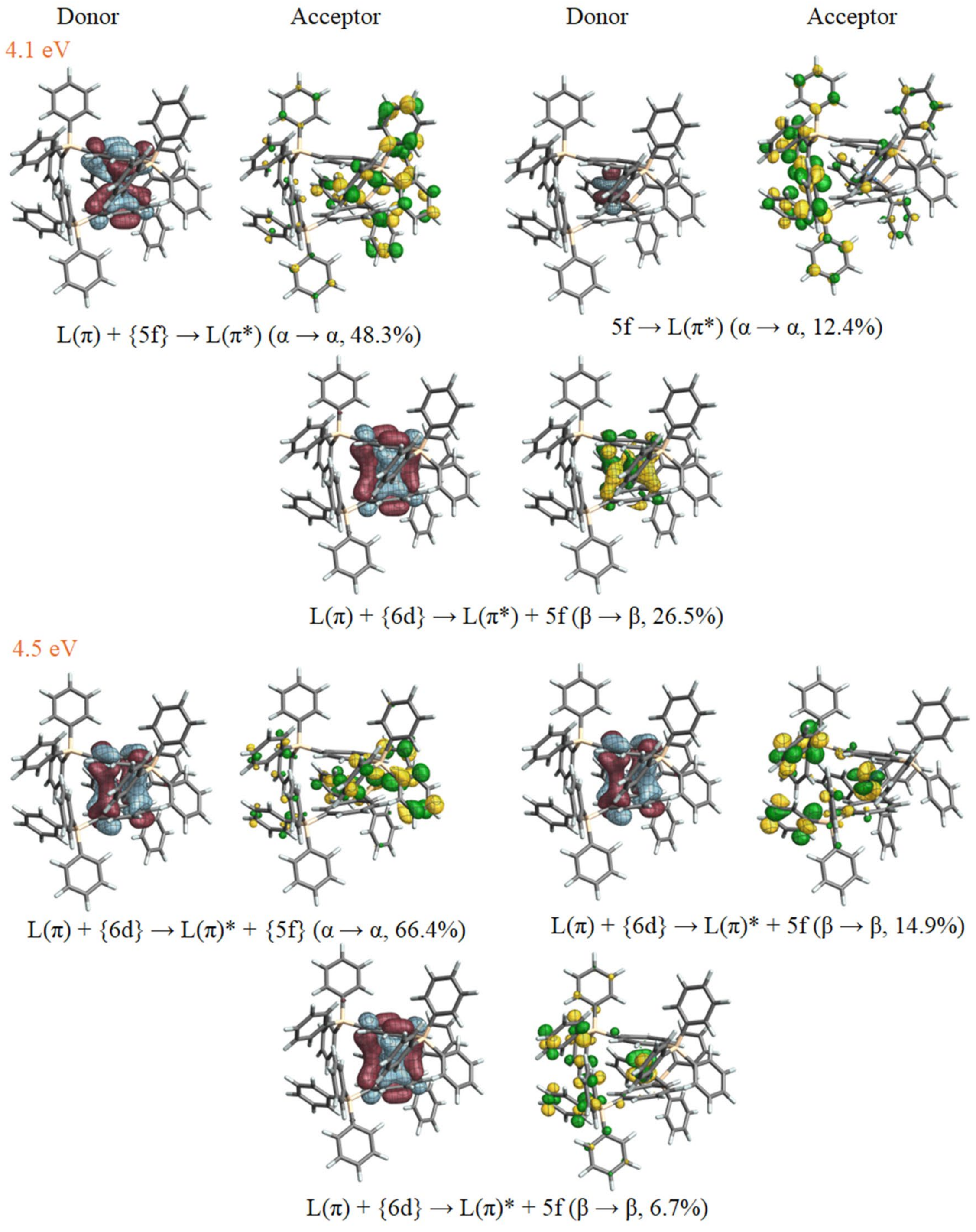

Figure S31. TPSSh calculations. Natural transition orbitals (NTOs, ±0.03 isosurfaces) corresponding to the important transitions in TD-DFT computed UV-Vis spectrum of **1U** in toluene covering from 3.5 to 5.0 eV. Weight-% of the NTOs corresponding to α→α and β→β transitions are listed. {} denotes a visible but small contribution. Transition energies given in the figure correspond to the calculations without applying a shift.

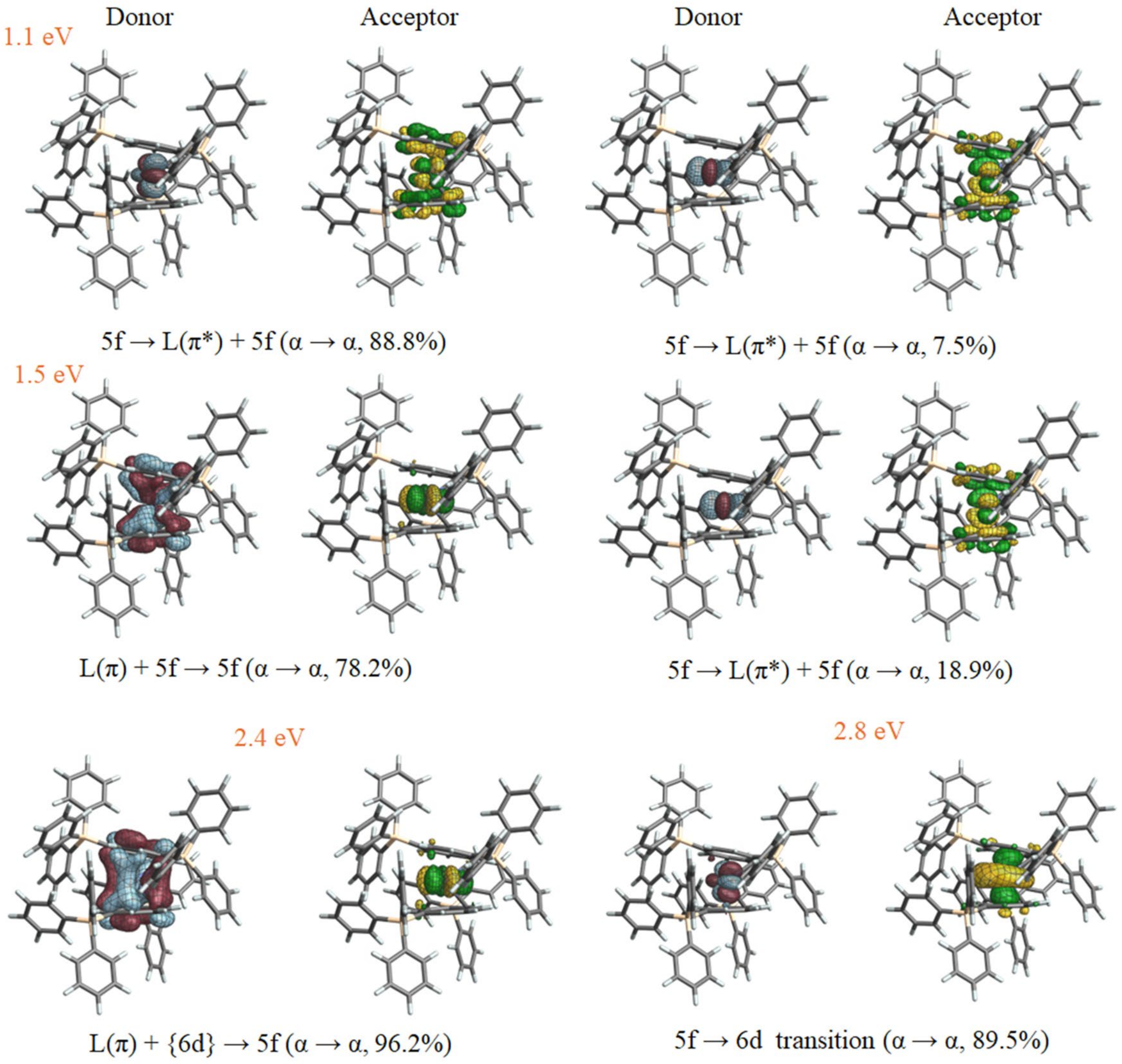

Figure S32. TPSSh calculations. Natural transition orbitals (NTOs, ±0.03 isosurfaces) corresponding to the important transitions in TD-DFT computed UV-Vis spectrum of **1Np** in toluene up to 2.5 eV. Weight-% of the NTOs corresponding to α→α and β→β transitions are listed. {} denotes a visible but small contribution. Transition energies given in the figure correspond to the calculations without applying a shift.

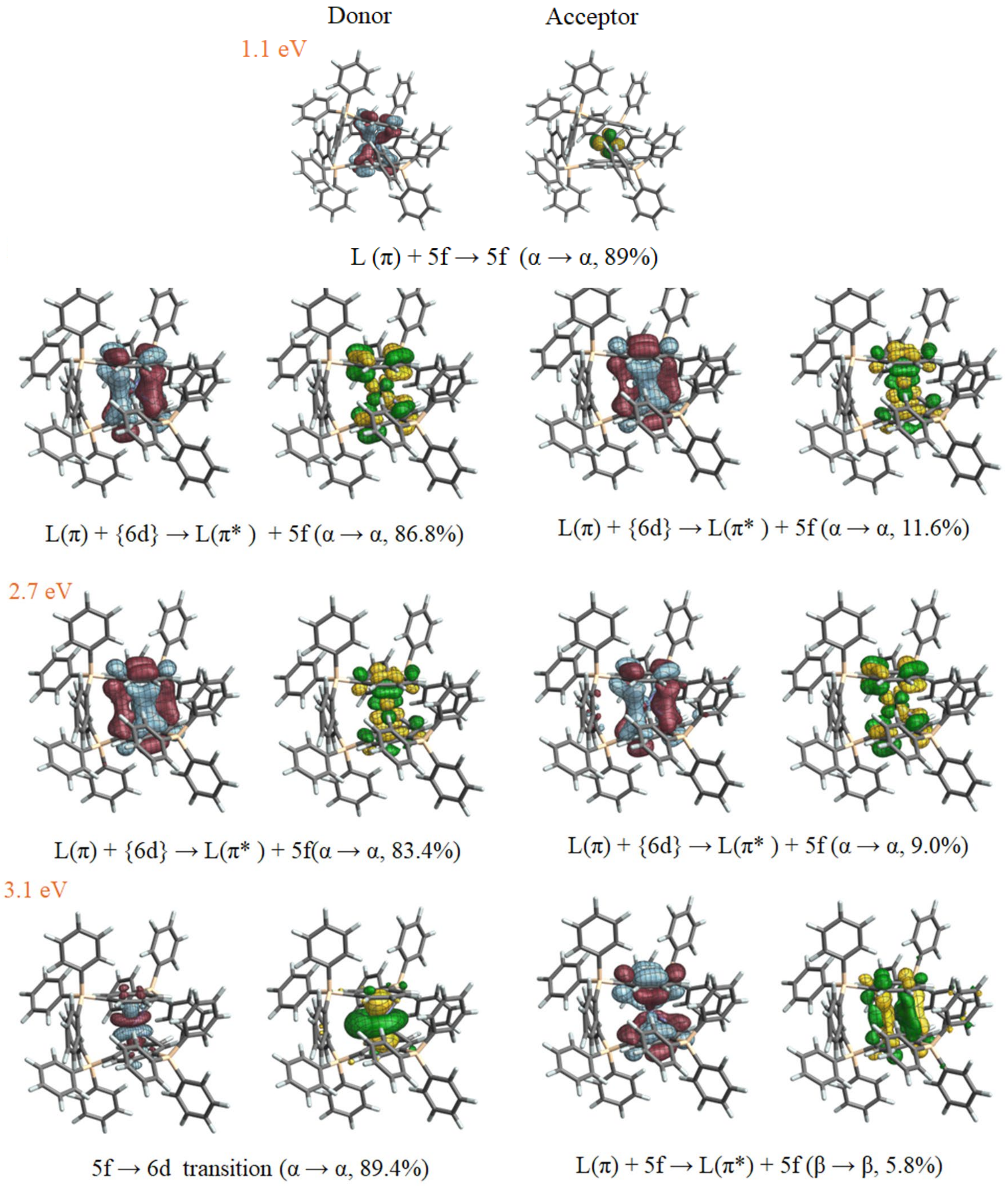

Figure S33. TPSSh calculations. Natural transition orbitals (NTOs, ±0.03 iso-surfaces) corresponding to the important transitions in TD-DFT computed UV-Vis spectrum of **1Pu** in toluene. Weight-% of the NTOs corresponding to α→α and β→β transitions are listed. {} denotes a visible but small contribution. Transition energies given in the figure correspond to the calculations without applying a shift.

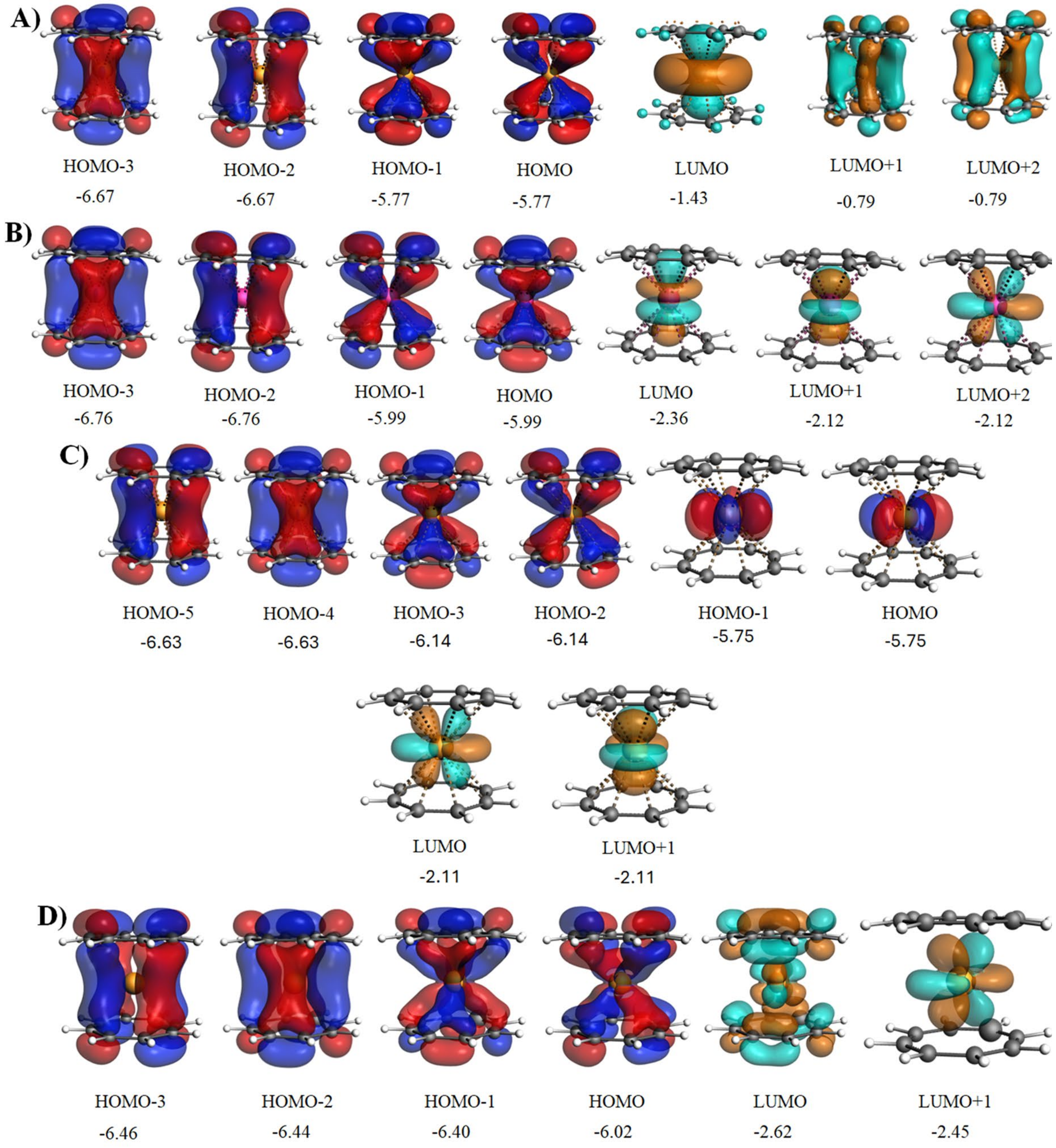

Figure S34. PBE0 calculations with toluene solvent model. Energies of FMOs (±0.03 iso-surfaces) in A) **Th(COT)₂**, B) **U(COT)₂**, C) **Np(COT)₂**, D) **Pu(COT)₂** complexes respectively. The canonical molecular orbitals shown here correspond to α-spin. Energies are in eV.

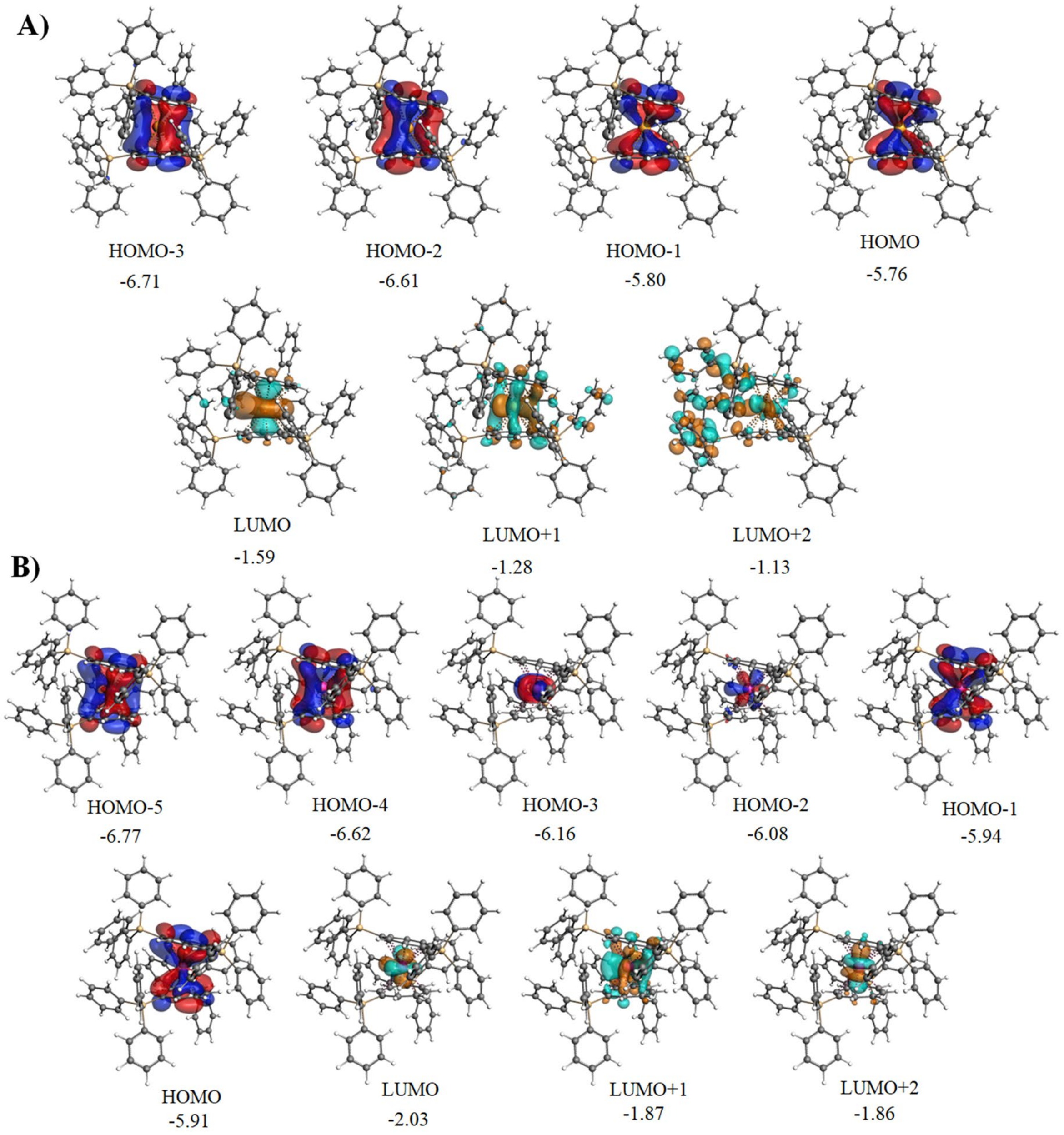

Figure S35. PBE0 calculations with toluene solvent model. Energies of FMOs (±0.03 iso-surfaces) in **1An** [An = Th (A)-U(B)] complexes. The canonical molecular orbitals shown here correspond to α-spin. Energies are in eV.

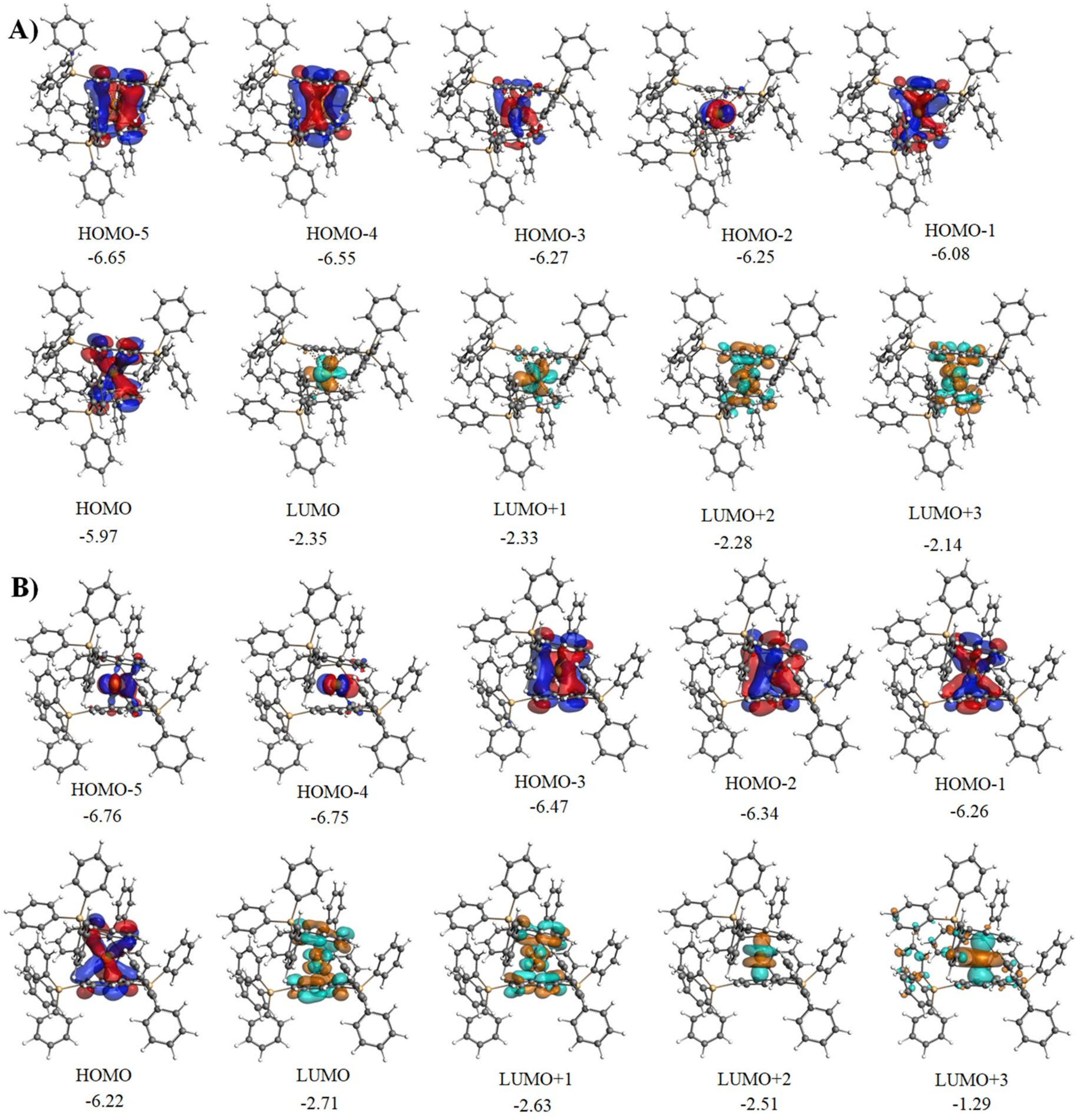

Figure S36. PBE0 calculations with toluene solvent model. Energies of FMOs (±0.03 iso-surfaces) in **1An** [An = Np (A)-Pu(B)] complexes. The canonical molecular orbitals shown here correspond to α-spin. Energies are in eV.

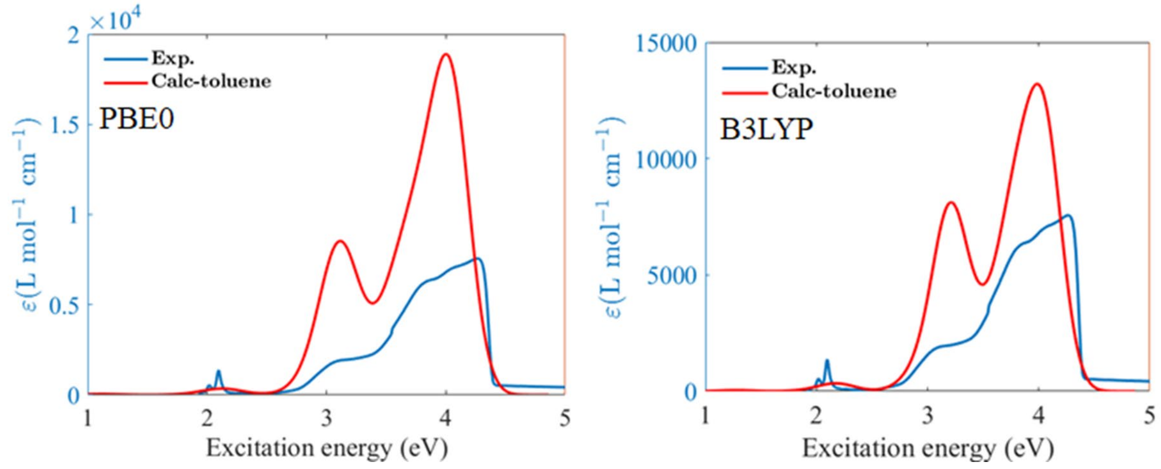

Figure S37. Overlay of experimentally recorded (red) with TDDFT computed (toluene solvent model, blue) UV-Vis spectrum of **1U** with PBE0 (left panel, red shift 0.6 eV) vs. B3LYP (right panel, red shift 0.3 eV). The Gaussian broadening parameter for the calculated spectra was σ = 0.15 eV.

S7. References

1. Sheldrick, G. M. SHELXT – Integrated space-group and crystal-structure determination. *Acta Cryst A* **71**, 3–8 (2015).
2. Dolomanov, O. V., Bourhis, L. J., Gildea, R. J., Howard, J. a. K. & Puschmann, H. OLEX2: a complete structure solution, refinement and analysis program. *J Appl Cryst* **42**, 339–341 (2009).
3. Sheldrick, G. M. Crystal structure refinement with SHELXL. *Acta Cryst C* **71**, 3–8 (2015).
4. Lorenz, V. *et al.* Unprecedented Bending and Rearrangement of f-Element Sandwich Complexes Induced by Superbulky Cyclooctatetraenide Ligands. *J. Am. Chem. Soc.* **133**, 1257–1259 (2011).
